# A pan-cancer analysis of thioredoxin-interacting protein as an immunological and prognostic biomarker

**DOI:** 10.1186/s12935-022-02639-2

**Published:** 2022-07-17

**Authors:** Xuxue Guo, Mei Huang, Haonan Zhang, Qianhui Chen, Ying Hu, Yan Meng, Changjie Wu, Chenge Tu, Yongfeng Liu, Aimin Li, Qingyuan Li, Peirong Zhou, Side Liu

**Affiliations:** 1 Guangdong Provincial Key Laboratory of Gastroenterology, Department of Gastroenterology, Nanfang Hospital, Southern Medical University, No. 1838, Guangzhou Avenue North, Guangzhou, 510515 Guangdong People’s Republic of China; 2 State Key Laboratory of Organ Failure Research, Guangdong Provincial Key Laboratory of Viral Hepatitis Research, Department of Infectious Diseases, Nanfang Hospital, Southern Medical University, Guangzhou, 510515 Guangdong People’s Republic of China; 3 Department of Critical Care, The First Affiliated Hospital of Gannan Medical University, Ganzhou, 341000 Jiangxi People’s Republic of China; 4 Department of Gastroenterology, The Third Affiliated Hospital of Guangzhou Medical University, Guangzhou, 510140 Guangdong People’s Republic of China

**Keywords:** TXNIP, Pan-cancer, Prognosis, Genetic alteration, Immune infiltration, Ubiquitination

## Abstract

**Background:**

The critical role of thioredoxin-interacting protein (*TXNIP*) in cellular sulfhydryl redox homeostasis and inflammasome activation is already widely known, however, no pan-cancer analysis is currently available.

**Methods:**

We thus first explored the potential roles of *TXNIP* across thirty-three tumors mainly based on The Cancer Genome Atlas and Gene Expression Omnibus datasets.

**Results:**

*TXNIP* is lowly expressed in most cancers, and distinct associations exist between *TXNIP* expression and the prognosis of tumor patients. *TXNIP* expression was associated with tumor mutational burden, microsatellite instability, mismatch repair genes, tumor infiltrating immune cell abundance as well as cancer-associated fibroblasts. Moreover, ubiquitin mediated proteolysis, protein post-translational modification and other related pathways were involved in the functional mechanisms of *TXNIP*.

**Conclusions:**

Our first pan-cancer study comprehensively revealed the carcinostatic role of *TXNIP* across different tumors. And this molecule may be considered as a potential immunological and prognostic biomarker.

**Supplementary Information:**

The online version contains supplementary material available at 10.1186/s12935-022-02639-2.

## Introduction

In view of the complex biological functions of *TXNIP*, it is necessary to carry out a multi-dimensional pan-cancer expression analysis of *TXNIP* and further assess its potential molecular mechanisms. We performed the *TXNIP* pan-cancer analysis mainly using the public TCGA (The Cancer Genome Atlas) and GEO (Gene Expression Omnibus) databases [1, 2].

**Fig. 1:**
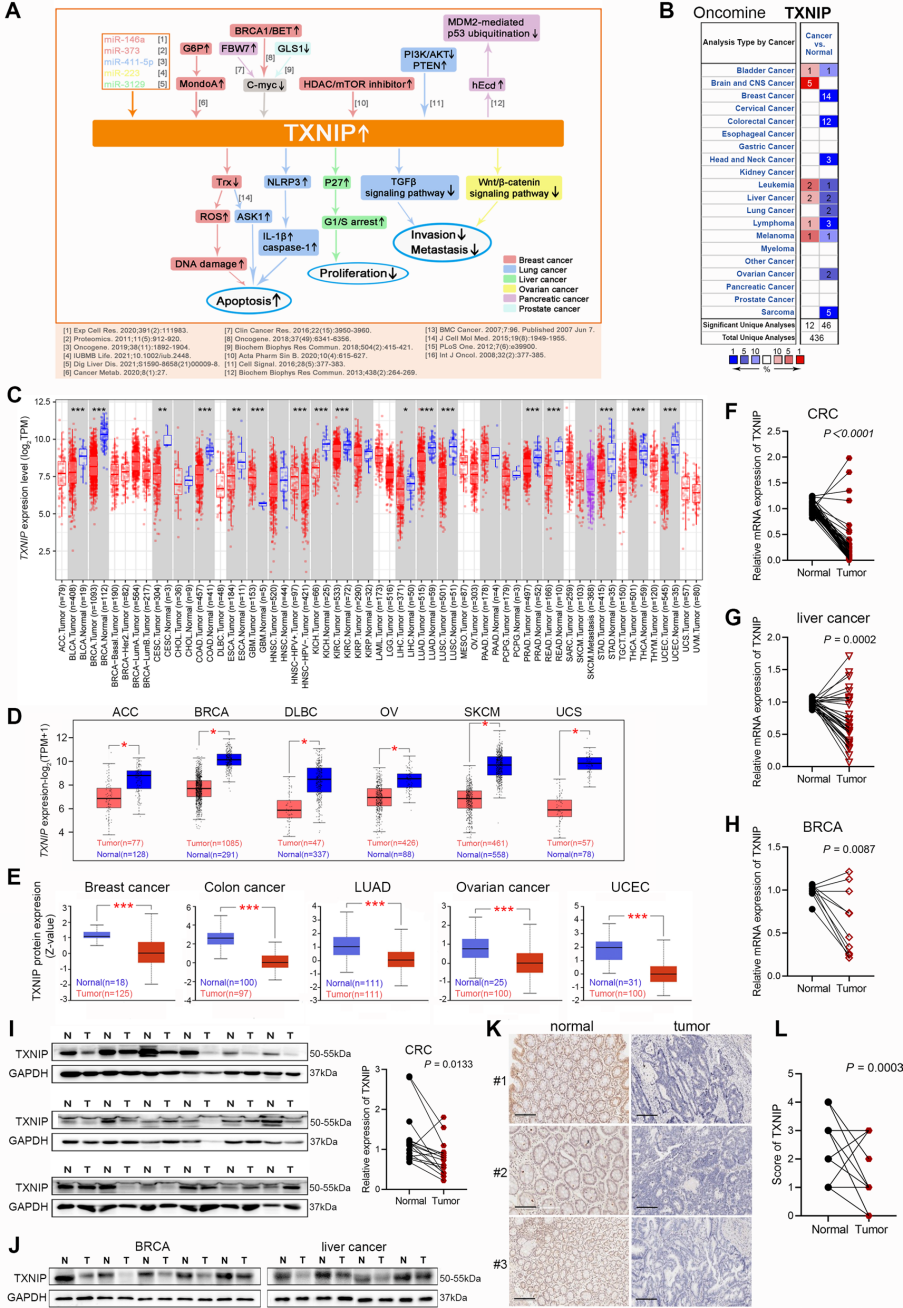
*TXNIP* expression in different types of human tumors. **A** Diagram for the reported association between TXNIP and various tumors. The reported pathways activated by TXNIP in different tumors are presented in a pictorial manner. The relevant references are also indicated. **B** Increased or decreased *TXNIP* in data sets of different tumors compared to normal tissues in the Oncomine database. The cell number represents the dataset number that meets all thresholds, red represents over-expression, and blue represents under-expression. **C** Expression level of *TXNIP* in different types of human tumors from TCGA data via TIMER2. **D** For ACC, DLBC, BRCA, OV, SKCM and UCS in the TCGA project, the corresponding normal tissues in GTEx database were setted as controls. **E** TXNIP total protein in normal tissue and primary tissue of breast cancer, colon cancer, ovarian cancer, LUAD and UCEC in CPTAC dataset. (*P < 0.05; **P < 0.01; ***P < 0.001). RT-qPCR analysis of the *TXNIP* mRNA expression in tumor specimens and matched normal tissues from patients with **F** CRC (n = 50), **G** liver cancer (n = 32) and **H** BRCA (n = 10). **I** Western blotting analysis of the TXNIP expression in 18 pairs of randomly selected CRC samples. **J** Western blotting analysis of the TXNIP expression in 5 pairs of BRCA and 4 pairs of liver cancer samples. **K** Representative IHC staining of TXNIP in cancer and adjacent normal tissue from CRC patients. Scale bars: 100 μm. **L** Immunohistochemical score of TXNIP

Thioredoxin-interacting protein (*TXNIP*), also known as Thioredoxin-binding protein-2 or Vitamin D3 up-regulated protein-1, was originally identified as an endogenous antagonist of thioredoxin (Trx), a key regulator in cellular redox equilibrium [3]. The *TXNIP* gene belongs to the arrestin family and structure analyses suggests that it is highly conserved among species [3, 4]. With regard to the human TXNIP protein, two alternatively spliced isoforms have been reported, and we have focused on TXNIP1 (NM_006472.6, isoform 1) for expression verification in this study. TXNIP protein may act as an oxidative stress mediator by inhibiting Trx activity or limiting its bioavailability, however this protein also exerts several other physiological and pathological effects, including regulating cell proliferation, cell division, apoptotic process, and cellular response to estradiol/progesterone or mechanical/chemical stimulus [5–7]. Importantly, the role of *TXNIP* in carcinogenesis and modulating tumor progression has been attracting increasing interest. Figure 1A provides an overview of the specific role of *TXNIP* in cancer. Unfortunately, our knowledge of the specific implication concerning *TXNIP* still remains limited. Unraveling the overall situation of the expression, mutation, immune response and prognostic potential of *TXNIP* is of great significance to grasp its essential role in cancer.

Here, we first utilized data from the TCGA and Genotype-Tissue Expression (GTEx) data portals to investigate the expression profiles of *TXNIP* in various types of cancer and paired normal tissues. And then, the survival status, genetic alteration as well as the immune infiltration of *TXNIP* in different cancers were identified. We also explored the potential molecular mechanism of *TXNIP* in the oncogenesis or clinical prognosis of malignancies by analyzing relevant protein ubiquitination sites, protein phosphorylation sites, and related cellular pathway.

## Materials and methods

### Gene expression analysis

The systematic analysis of *TXNIP* expression levels between tumor and adjacent normal tissues was conducted according to RNA sequencing data from the TCGA project via the “Gene_DE” functional module of the Tumor Immune Estimation Resource 2 (TIMER2) web (http://timer.cistrome.org) [8]. Gene Expression Profiling Interactive Analysis 2 (GEPIA2) (http://gepia2.cancer-pku.cn/#analysis) [9] was applied to profile the tissue-wise expression of *TXNIP* gene in several cancer types without normal or with highly limited normal tissues [e.g., adrenocortical carcinoma (ACC), lymphoid neoplasm diffuse large B-cell lymphoma (DLBC), ovarian serous cystadenocarcinoma (OV), etc.]. The threshold settings were as follows: | log_2_FC (fold change) | cutoff = 1, *P*-value cutoff = 0.01, and “Match TCGA normal and GTEx data”. Additionally, the differential expression between tumor tissues and the matched normal tissues was also identified in the Oncomine database (https://www.oncomine.org) [10]. We identified the expression levels of the total protein of TXNIP (NP_006463.3) in UALCAN portal (http://ualcan.path.uab.edu/analysis-prot.html) [11].

### Clinical specimens

Patients who underwent radical operation for CRC (n = 50), liver cancer (n = 32) and BRCA (n = 10) in our hospital were included in this study. All patients obtained informed consent and none of them had received any radiotherapy or chemotherapy before surgery. Each patient sample was confirmed by histopathology. Fifty pairs of cancer tissues and matched normal tissues were prepared for qRT-PCR analysis, of which 22 pairs were randomly selected for immunohistochemistry (IHC) analysis, and 18 pairs for western blotting analysis. The expression of *TXNIP* at mRNA levels was also detected in 32 pairs of liver cancer and 10 pairs of BRCA tumor tissues with their adjacent normal tissues by RT-qPCR. Five pairs of BRCA and 4 pairs of liver cancer samples were prepared for western blotting analysis. The experimental protocol was approved by the Human Subjects Protection Committee of Nanfang Hospital (NFEC-201809-K3).

### Survival prognosis analysis

“Survival” module of GEPIA2 was utilized to assess the correlation between *TXNIP* expression and prognosis of cancers. The overall survival (OS) and disease-free survival (DFS) significance map data of *TXNIP* was obtained using Mantel-Cox test, under the settings of cutoff-high = 50%, cutoff-low = 50%, group cutoff = Median, and significance level = 0.05.

The Kaplan Meier plotter database (http://kmplot.com/analysis/) [12] is applied to assess the impact of different genes on the survival rate of multiple malignancies. To analyze the prognostic value of *TXNIP*, the patient samples are split into two groups on the bases of the quantile expressions of *TXNIP*. Then, the two patient cohorts are compared by a Kaplan–Meier survival plot, and the hazard ratio (HR) with 95% confidence intervals (CI) and logrank *P*-value are calculated. A *P*-value < 0.05 was considered statistically significant.

### Genetic alteration analysis

Data concerning the alteration frequency of *TXNIP* gene and the schematic diagram of the mutated site information of *TXNIP* were acquired in the “Cancer Types Summary” and “Mutations” module in cBioPortal (http://cbioportal.org) [13].

### Correlation analysis of TXNIP and TMB/MSI/MMR/DNMT/PTM

The online Sangerbox tools (http://sangerbox.com/tool) was used to evaluate the potential correlation between *TXNIP* expression and the tumor mutational burden (TMB) or microsatellite instability (MSI) in different TCGA cancer cases. Spearman’s rank correlation test was performed, and the *P*-value and partial correlation (cor) value were obtained. Data revealing the relationship of *TXNIP* with the five Mismatch repair (MMR) genes (MLH1, MSH2, MSH6, PMS2, and EPCAM) and four DNA methyltransferases (DNMTs) in all TCGA tumors was generated through the same public platform.

Protein post-translational modification (PTM) regulates a broad range of cellular biological processes, many of which are closely related to multiple cancer types [14–16]. We analyzed the protein modification sites of TXNIP using the open-access SMART web (http://smart.embl.de/), and obtained the predicted ubiquitination and phosphorylation features of TXNIP.

### Immune infiltration analysis

The *TXNIP* expression in different TCGA tumors and its correlation with the abundances of eight tumor-infiltrating immune cells (TIICs), together with cancer-associated fibroblasts (CAFs) was explored by the corresponding gene modules of TIMER2 web server. The *P*-values and partial cor values were obtained via the purity-adjusted Spearman’s rank correlation test. Additionally, the correlation module was applied to infer the relationships between *TXNIP* expression and gene markers of TIICs in three given cancer types, and a total of 62 immune-related gene markers were selected for analysis via the CellMarker database (http://bio-bigdata.hrbmu.edu.cn/CellMarker/) [17].

For reliable evaluation of tumor immune microenvironment, we used an R software immuneeconv to analyze the immune scores of each tumor sample. Immune checkpoint inhibitor is a promising therapy for tumor immunotherapy [18]. The expression values of the 47 transcripts related to immune checkpoints was extracted, and its association with *TXNIP* expression in different tumor tissues was organized in a heat map. The expression levels of immune checkpoints genes in the same tumor tissue was averaged first, and then performed zscore standardization. Tumor neoantigen is a new antigen encoded by mutated genes of tumor cells, which has immunological activity [19]. Therefore, it is expected to design and synthesize therapeutic vaccines for specific mutations of tumor cells. Herein we collected the numbers of neoantigen in multiple cancers and further analyzed the relationship between *TXNIP* expression and the numbers of neoantigen of each type of tumor.

### TXNIP-related gene enrichment analysis

The interaction network of the experimentally verified TXNIP-binding proteins were obtained via the STRING website (https://string-db.org/). The top 100 *TXNIP*-correlated targeting genes was acquired using the GEPIA2 web server based on the datasets of all TCGA tumor and normal tissues. Further, the pairwise gene Pearson correlation analysis of *TXNIP* and selected genes was conducted by GEPIA2, and the log_2_ TPM was applied for the dot plot. The heatmap data of the abovementioned genes was supplied by the “Gene_Corr” module of TIMER2, which contained the partial Cor and *P*-value in the purity-adjusted Spearman’s rank correlation test. Moreover, the Jvenn (http://bioinformatics.psb.ugent.be/webtools/Venn/), a practical tool for drawing custom Venn diagrams of list of elements, was used to calculate the intersection of the *TXNIP*-binding and interacted genes.

Moreover, the Kyoto Encyclopedia of Genes and Genomes (KEGG) pathway analysis was performed with the combined gene list. The functional annotation chart of the *TXNIP* related genes was obtained from the Database for Annotation, Visualization, and Integrated Discovery (DAVID) database (https://david.ncifcrf.gov/home.jsp) [20]. To conduct the Gene Ontology (GO) enrichment analysis, data for biological process (BP), cellular component (CC), and molecular function (MF) were visualized as circular plots through the RCircos R package. The R software v4.0.3 (https://www.r-project.org/) was used in this analysis.

### RNA isolation and quantitative real-time PCR (qRT-PCR)

Total RNA was isolated from tissues using the TRIzol reagent (TaKaRa, China). RT-qPCR was performed using the PrimeScript RT Reagent Kit (#RR035A, TaKaRa, China) and SYBR Premix Ex Taq (#RR820A, TaKaRa, Dalian, China) according to the manufacturer’s instructions. Bio-software Primer Premier 5.0 was applied to design primers, and the specific primers used are as follows: TXNIP-forward: ATTGGAGAGCCCAACCACTC, TXNIP-reverse: TTCCACATGCTCACTGCACA, GAPDH-forward: AAATCCCATCACCATCTTCC, GAPDH-reverse: TCACACCCATGACGAACA. The RT-qPCR results were analyzed to obtain the Ct values of the amplified products, and data were analyzed by the 2^−ΔΔCt^ method.

### Western blotting

Western blotting was conducted as previously described [21]. Protein lysates were prepared, subjected to 10% sodium dodecyl sulfate polyacrylamide gel electrophoresis (SDS-PAGE), transferred onto polyvinylidene difluoride (PVDF) membranes and blotted according to standard methods using the following antibodies: TXNIP (1:1000, 18243-1-AP, Proteintech), GAPDH (1:50,000, 60004-1-Ig, Proteintech).

### TXNIP IHC analysis

IHC staining was performed following the manufacturer’s instructions (PV-6001, ZSGB-BIO, Beijing, China) using TXNIP (1:200, 18243-1-AP, Proteintech). Two independent pathologists used software ImageJ to calculate the proportion of positive area. Data were expressed as the average of three randomly selected microscopic fields.

### Statistical analysis

Gene expression data from the TCGA and GTEx databases were analyzed using Student’s t-test. In PrognoScan, the univariate Cox regression model was used to calculate the HR and *P* value. In GEPIA2 and Kaplan–Meier Plotter, log rank test was used to calculate the HR and its *P* value in order to compare survival curves. The correlations between *TXNIP* expression and abundance scores of immune cells/TMB/MSI/MMR/DNMT evaluated by Spearman’s correlation. Results with *P* < 0.05 were considered as statistically significant, providing credibility for the data analysis.

## Results

### TXNIP expression analysis data

To conduct a comprehensive analysis of human *TXNIP* (NM_006472.6 for mRNA, NP_006463.3 for protein, data relating with its genome location, conserved functional domain and the phylogenetic tree were obtained (Additional file 1: Fig. S1A–C). As shown in Additional file 1: Fig. S1D, the levels of mRNA expression of *TXNIP* shows a low tissue specificity with all normalized expression values in detected tissues > 1. Then, a low cell type specificity of *TXNIP* expression in different blood cells is also observed (Additional file 1: Fig. S1E).

To figure out whether TXNIP expression correlates with cancer, we firstly evaluated TXNIP expression in different tumors and adjacent normal tissues using the online Oncomine database. The results showed that TXNIP expression was lower in several cancer groups than in normal tissues, including breast cancer, colorectal cancer (CRC), head and neck cancer, lung cancer, ovarian cancer, sarcoma (SARC), lymphoma, liver cancer or bladder cancer (Fig. 1B, all *P* < 0.05), and more detailed results were summarized in Additional file 8: Table S1. Moreover, we further used TIMER2 and GEPIA2 web servers to evaluate the RNA sequencing data of TXNIP in TCGA and GTEx. Data confirmed that significantly more tumor tissues expressed lower levels of *TXNIP* mRNA than the corresponding control tissues (Fig. 1C, D), of which included BLCA, UCEC, breast invasive carcinoma (BRCA), LUAD, lung squamous cell carcinoma (LUSC), colon adenocarcinoma (COAD), rectum adenocarcinoma (READ), stomach adenocarcinoma (STAD), kidney chromophobe (KICH), kidney renal clear cell carcinoma (KIRC), prostate adenocarcinoma (PRAD), thyroid carcinoma (THCA) (*P* < 0.001), cervical squamous cell carcinoma and endocervical adenocarcinoma (CESC), esophageal carcinoma (ESCA) (*P* < 0.01), OV, DLBC, ACC, skin cutaneous melanoma (SKCM), uterine carcinosarcoma (UCS), and liver hepatocellular carcinoma (LIHC) (*P* < 0.05). Results of the TXNIP proteomic expression profile analysis showed absolutely lower expression of TXNIP total protein in the primary tissues of breast cancer, colon cancer, ovarian cancer, LUAD and UCEC (Fig. 1E, all *P* < 0.001) than in normal tissues. Taken together, the data confirmed that the *TXNIP* gene was down-regulated in multiple cancers compared to normal samples.

To verify the above analysis results, we collected fresh tumor specimens and matched normal tissues from patients with CRC, BRCA and liver cancer in our hospital. The qPCR analysis revealed that the relative expression level of *TXNIP* mRNA in CRC (n = 50) was significantly lower than that in adjacent tissues (Fig. 1F, *P* < 0.0001). And moreover, 22 of 32 pairs of liver cancer tissues displayed significantly lower transcriptional level of *TXNIP* than that in paired margin tissues, and 7 of 10 pairs of BRCA (Fig. 1G, H, *P* = 0.0002 and *P* = 0.0087, respectively). Further, western blotting analysis of 18 pairs of randomly selected CRC samples showed that tumor tissues exhibited markedly lower level of TXNIP than the adjacent tissues (Fig. 1I, *P* = 0.0133). Simultaneously, similar results were obtained in the BRCA and liver cancer samples (Fig. 1J). TXNIP IHC staining of 22 pairs of CRC samples drew consistent conclusion. TXNIP was lowly expressed in 12 out of 22 tumor tissues and highly expressed in 19 out of 22 normal tissues (Fig. 1K). Immunohistochemical score of TXNIP showed that the expression level of TXNIP in tumor tissues was significantly lower than that in paired normal tissues (Fig. 1L, *P* = 0.0003).

### Survival analysis data

**Fig. 2:**
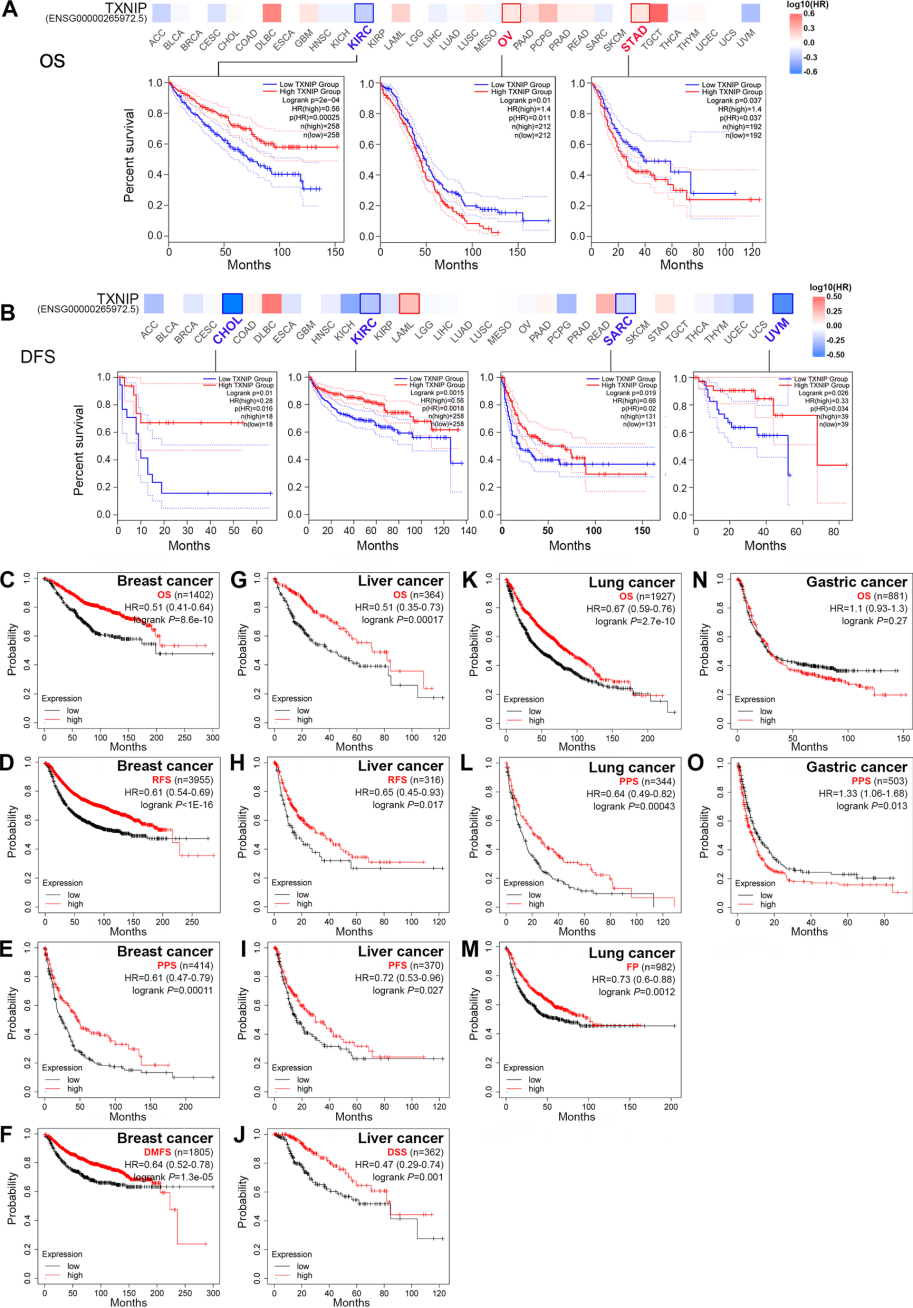
Survival curves comparing the high and low expression of TXNIP in different tumors. **A** OS analyses in GEPIA2. **B** DFS analyses in GEPIA2. **C**–**F** OS, RFS, PPS and DMFS survival curves of breast cancer in Kaplan–Meier plotter databases (n = 1402, n = 3955, n = 414, and n = 1805, respectively). **G**–**J** OS, RFS, PFS and DSS survival curves of liver cancer in Kaplan Meier plotter databases (n = 364, n = 316, n = 370, and n = 362, respectively). **K**–**M** OS, PPS and FP survival curves of lung cancer in Kaplan Meier plotter databases (n = 1927, n = 344, and n = 982, respectively). **N**, **O** OS and PPS survival curves of gastric cancer in Kaplan Meier plotter databases (n = 881 and n = 503, respectively). *HR* hazard ratio, *CI* confidence interval, *OS* overall survival, *DFS* disease-free survival, *RFS* relapse-free survival, *PPS* post-progression survival, *PFS* progression-free survival, *DSS* disease-specific survival, *DMFS* distant metastasis-free survival, *FP* first progression

Next, we analyzed the prognostic value of TXNIP expression across cancers in GEPIA2. As shown in Fig. 2A, OS analysis data showed a correlation between low *TXNIP* expression and poor prognosis for cancers of KIRC (*P* = 2.0e−04) within the TCGA project. Meanwhile, lowly expressed *TXNIP* was linked to poor prognosis of DFS for the TCGA cases of CHOL (*P* = 0.010), KIRC (*P* = 0.002), SARC (*P* = 0.019), and uveal melanoma (UVM, *P* = 0.026) (Fig. 2B). Interestingly, high expression of the *TXNIP* gene was related to poor OS prognosis for OV and STAD (Fig. 2A, all *P* < 0.05), suggesting the prognostic value of *TXNIP* may has tissue or tumor specificity.

Moreover, the *TXNIP* prognostic value was evaluated by the Kaplan–Meier plotter database using Affymetrix microarrays data (Fig. 2C–O, Additional file 2: Fig. S2A–H). And results demonstrated that low levels of *TXNIP* was closely linked to poor OS, relapse-free survival (RFS), post-progression survival (PPS), and distant metastasis-free survival (DMFS) prognosis for breast cancer (Fig. 2C–F, all *P* < 0.001); poor OS (*P* = 0.000), RFS (*P* = 0.017), progression-free survival (PFS, *P* = 0.027), and disease specific survival (DSS, *P* = 0.001) prognosis for liver cancer (Fig. 2G–J); and poor OS (*P* = 2.7e−10), PPS (*P* = 0.000), and first progression (FP, *P* = 0.001) prognosis for lung cancer (Fig. 2K–M). However, we failed to detect a correlation between *TXNIP* expression and the OS, PFS and PPS prognosis of ovarian cancer (data not shown, all *P* > 0.05). Based on the datasets of the Kaplan–Meier plotter, we observed that high expression of *TXNIP* was associated with poor clinical outcomes of OS and PPS for gastric cancer, especially in subgroup analysis of “stage 2 to 4”, “lauren classification/intestinal/diffuse” and “treatment/surgery alone” (Additional file 8: Table S2). We also analyzed the correlation of *TXNIP* expression and the selected clinicopathological factors in liver (Additional file 8: Table S3), lung (Additional file 8: Table S4), and breast cancer (Additional file 8: Table S5), and finally observed distinct conclusions concerning these tumors. These findings suggest that low *TXNIP* expression implies reduced survival in breast, lung and liver cancer.

In addition to *TXNIP* microarray analysis in the Kaplan–Meier plotter database, the impact of *TXNIP* expression to survival rates of patients was evaluated using the PrognoScan (Additional file 2: Fig. S2I–T and Additional file 8: Table S6). Notably, decreased expression of *TXNIP* indicated poorer survival prognosis in seven out of eight cancers, including breast, bladder, skin, lung, brain, eye and soft tissue cancers (Additional file 2: Fig. S2I–S, all *P* < 0.05). Unexpectedly, analysis of two cohorts (GSE14333, GSE17536) showed that high *TXNIP* expression was linked to poorer DFS prognosis of CRC patients (Additional file 2: Fig. S2T and Additional file 8: Table S6, all *P* < 0.05). Accordingly, it is conceivable that low *TXNIP* expression is an independent risk factor and leads to a poor prognosis in breast, bladder, and lung cancer patients.

### Genetic alteration analysis data

**Fig. 3:**
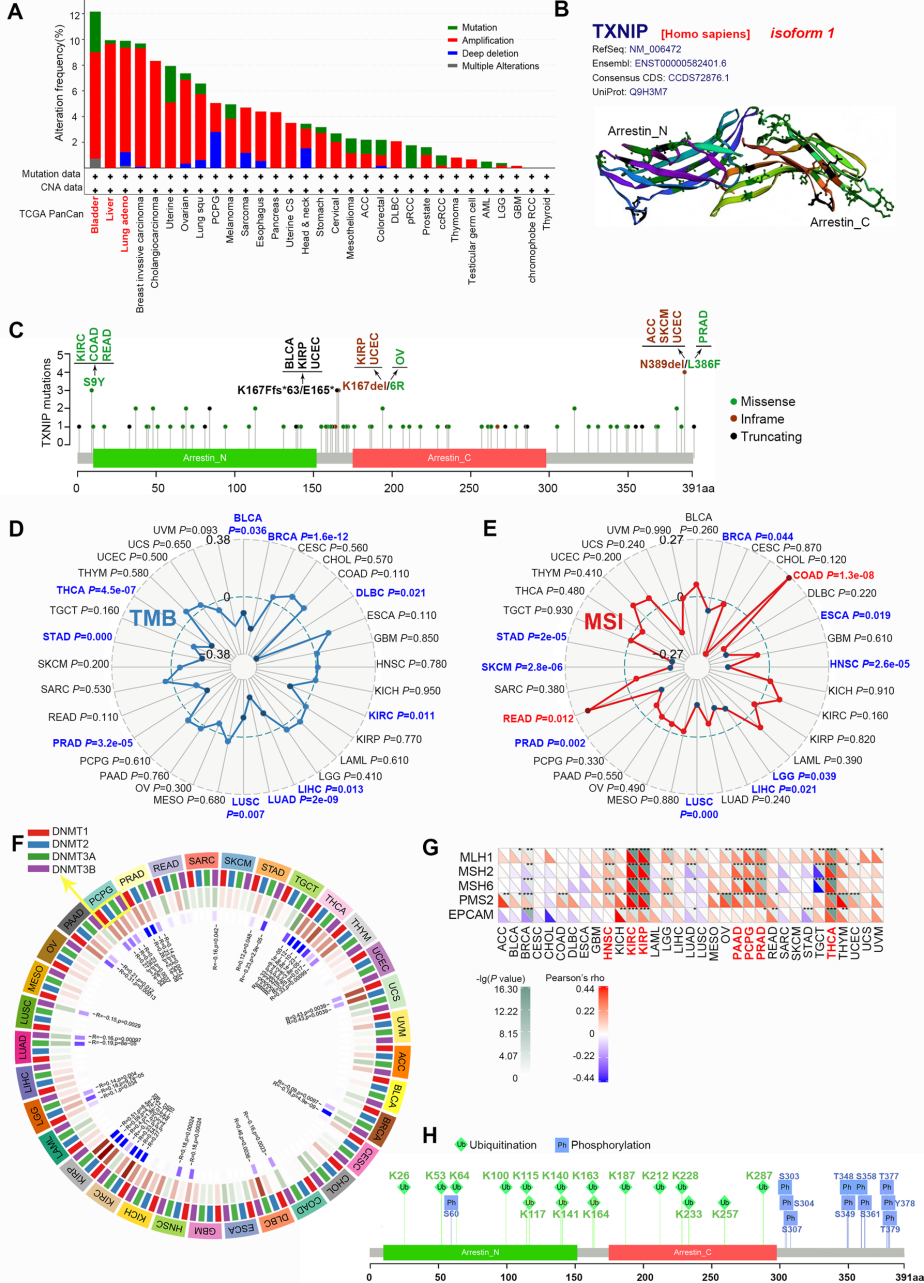
Mutation and modification feature of *TXNIP* in different TCGA tumors. **A** The genetic alteration type and frequency of TXNIP in various cancers. **B** The three-dimensional structure of *TXNIP* protein. **C** Mutation site of the *TXNIP*. **D** Correlation analysis of tumor mutational burden (TMB) with *TXNIP* gene expression. **E** Correlation analysis of microsatellite instability (MSI) with *TXNIP* gene expression. **F** Correlation analysis of four DNA methyltransferases (DNMT1, DNMT2, DNMT3A, DNMT3B) with *TXNIP* gene expression. **G** Correlation analysis of five MMR genes (EPCAM, MLH1, MSH2, MSH6 and PMS2) with *TXNIP* gene expression. **H** The predicted ubiquitination and phosphorylation sites of TXNIP protein

The genetic alteration status of *TXNIP* in different tumor samples of the TCGA cohorts was examined by the cBioPortal website. As shown in Fig. 3A, bladder (n = 411), liver (n = 372) and lung (n = 566) cancer contribute to the top 3 tumors with the highest alteration frequencies of *TXNIP*, with an alteration frequency of 12.17%, 9.95%, and 9.89%, respectively. It is worth noting that the “amplification” type of copy number aberration (CNA) is the primary genetic alteration type in almost all tumors. The three-dimensional structure of TXNIP protein as well as the sites and types of the *TXNIP* genetic alteration are further presented in Fig. 3B, C. The missense mutation of *TXNIP* appears to be the main type of genetic alteration in PRAD (Fig. 3C). N389del/L386F alteration, detected in ACC, SKCM, UCEC and PRAD (Fig. 3C), is able to induce inframe and missense mutation of the *TXNIP* gene, leading to the subsequent deletion of N (asparagine) at the 389 site of TXNIP protein, the substitution of L (Leucine) with F (Phenylalanine) at the 386 site of TXNIP protein, respectively.

The tumor mutational burden (TMB) is a quantitative biomarker, which can reflect the number of mutations in tumor cells [22]. Microsatellite instability (MSI), a mode of genomic instability, is closely related with carcinogenesis, prognosis and response to immunotherapy [23, 24]. To assess the role of TXNIP in tumorigenesis, we analyzed the correlation between TXNIP expression and TMB/MSI across all tumors of TCGA. As shown in Fig. 3D, we observed a negative correlation between *TXNIP* expression and TMB for BRCA (*P* = 1.6e−12), LIHC (*P* = 0.013), LUSC (*P* = 0.007), PRAD (*P* = 3.2e−05), BLCA (*P* = 0.036), DLBC (*P* = 0.021), KIRC (*P* = 0.011), LUAD (*P* = 2e−09), STAD (*P* = 0.000), and THCA (*P* = 4.5e−07). *TXNIP* expression is also negatively correlated with MSI of BRCA (*P* = 0.044), LIHC (*P* = 0.021), LUSC (*P* = 0.000), PRAD (*P* = 0.002), ESCA (*P* = 0.019), SKCM (*P* = 2.8e−06), STAD (*P* = 2e−05), head and neck aquamous cell carcinoma (HNSC) (*P* = 2.6e−05), brain lower grade glioma (LGG) (*P* = 0.039) but is positively correlated with that of COAD (*P* = 1.3e−08) and READ (*P* = 0.012) (Fig. 3E). This suggests that TXNIP may be associated with tumorigenesis and treatment.

The biological role of DNA methylation has been extensively studied. As a common epigenetic modification, its regulation is reported to be closely related to tumorigenesis [25]. Consequently, we next investigated the correlation between *TXNIP* expression and that of four epigenetic regulators (DNMT2, DNMT1, DNMT3A, and DNMT3B) via the Sangerbox platform. The expression of *TXNIP* is negatively correlated with that of DNMTs in KIRC, KIRP, PRAD and PCPG (all *P* < 0.05). The deficiency in mismatch repair (MMR), a common DNA repair pathway, may increase mutations and result in MSI and carcinogenesis [13]. Furthermore, *TXNIP* expression and the levels of five MMR genes, especially MLH1, MSH2, MSH6 and PMS2, are positively correlated in KIRC, kidney renal papillary cell carcinoma (KIRP), THCA, HNSC, PRAD, pancreatic adenocarcinoma (PAAD) and pheochromocytoma and paraganglioma (PCPG) (Fig. 3G, all *P* < 0.01). These results conformably demonstrate the important role of *TXNIP* as a tumor suppressor in certain types of cancer, suggesting that its deficiency may play a crucial role in oncogenesis.

Ubiquitination and phosphorylation are the enzymatic post-translational protein modification that regulate cellular biological processes in various ways and have been implicated in a number of cancer types [14–16]. It is of note that dysregulation of specific protein ubiquitination may contribute substantially to cancer development and metastasis [16]. Here we outlined the possible ubiquitination and phosphorylation sites of TXNIP protein, as shown in Fig. 3H. This observation merits further molecular assays for further exploration, which may explain the mechanism of low expression of TXNIP protein.

### Immune infiltration analysis data

**Fig. 4:**
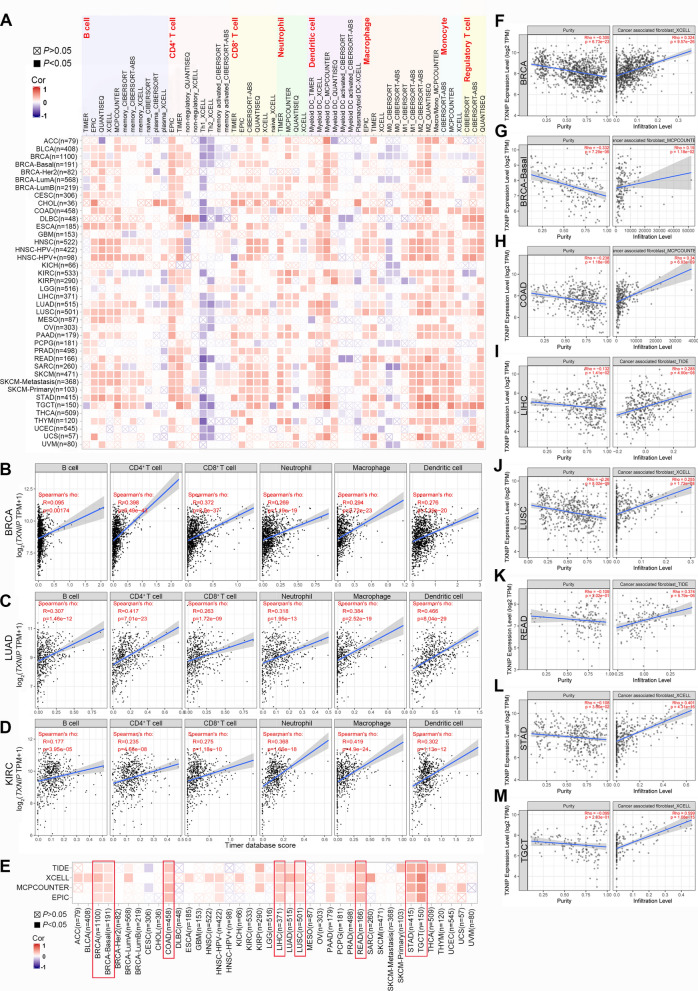
Correlation of TXNIP expression with immune infiltration level in different tumors of TCGA. **A** Correlation of *TXNIP* expression with infiltration level of B cell, CD4^+^ T cell, CD8^+^ T cell, neutrophil, dendritic cell, macrophage, monocyte, and regulatory T lymphocyte. **B**–**D** *TXNIP* expression was significantly positively related to the levels of infiltrating B cell, CD4^+^ T cell, CD8^+^ T cell, neutrophil, macrophage and dendritic cell in BRCA, LUAD and KIRC. **E** Correlation of *TXNIP* expression with immune infiltration of cancer-associated fibroblast. **F**–**M** *TXNIP* expression has significant negative correlations with tumor purity but was significantly positively correlated with the levels of infiltrating cancer-associated fibroblast in BRCA, BRCA-Basal, COAD, LIHC, LUSC, READ, STAD, and TGCT. *BRCA* breast invasive carcinoma, *LUAD* lung adenocarcinoma, *KIRC* kidney renal clear cell carcinoma, *COAD* colon adenocarcinoma, *LIHC* liver hepatocellular carcinoma, *LUSC* lung squamous cell carcinoma, *READ* rectum adenocarcinoma, *STAD* stomach adenocarcinoma, *TGCT* testicular germ cell tumors

Tumor-infiltrating immune cells, an indication of the host immune reaction to tumor antigens, play a critical role in tumor progression and antitumor activity [26–28]. As the major TIIC population, tumor infiltrating lymphocytes are independent predictors of sentinel lymph node status and cancer survival [27–29]. As shown in the heatmap of Fig. 4A, *TXNIP* expression was significantly correlated with TIICs in various types of cancers. Specifically, in the tumors of BRCA-LumA (n = 568), CESC (n = 306), ESCA (n = 185), HNSC (n = 522), HNSC-HPV- (n = 422), HNSC-HPV+ (n = 98), KIRC (n = 533), LIHC (n = 371), SARC (n = 260), SKCM (n = 471), and STAD (n = 415), *TXNIP* expression is negatively correlated with non-polarized M0 macrophages, but positively correlated with polarized M1 and M2 macrophages, especially the M2. We thus hypothesized that *TXNIP* may activate the tumor-associated macrophages (TAMs) along with shifting macrophage phenotype to a more anti-inflammatory state. Among these studied cancers, *TXNIP* expression has significant correlations with infiltrating levels of CD4^+^ T cells in 22 types of cancer, CD8^+^ T cells in 19 types of cancer, macrophages in 22 types of cancer, neutrophils in 25 types of cancer, and dendritic cells in 23 types of cancer (Fig. 4B–D and Additional file 3: Fig. S3, Additional file 4: Fig. S4, Additional file 5: Fig. S5 and Additional file 6: Fig. S6 all *P* < 0.05).

We then selected the specific cancers in which *TXNIP* was correlated with oncologic outcomes and infiltrating immune cells. Using the prognostic results related to *TXNIP* from the GEPIA2, Kaplan–Meier-plotter and PrognoScan analyses, we eventually selected BRCA, LUAD and KIRC for further research on immune infiltration via TIMER2. The *TXNIP* expression had significant positive correlations with six types of infiltrating immune cells in BRCA (Fig. 4B, all *P* < 0.01), LUAD (Fig. 4C, all *P* < 0.001), and KIRC (Fig. 4D, all *P* < 0.001). Tumor purity, defined as the proportion of tumor cells in the tumor sample, was reported to influence the genomic sequencing analysis of immune infiltration in clinical tumor samples [30]. After purity adjustment, we found that the correlation of TAMs genes (CCL2, CCL5 and CD68) obviously changed, especially the values in BRCA (n = 1100), which were the most significant changes (Additional file 8: Table S7). These findings strongly demonstrate that *TXNIP* could recruit immune cells in the TME in BRCA, LUAD and KIRC.

CAFs in the stroma of the TME were reported to participate in modulating the function of various TIICs [30]. As shown in Fig. 4E, we observed a statistical positive correlation of *TXNIP* expression and the estimated infiltration value of CAFs for the TCGA tumors of BRCA, BRCA-Basal, COAD, LIHC, LUSC, READ, STAD, and testicular germ cell tumors (TGCT) (all *P* < 0.05). The scatterplot data of the above tumors are presented in Fig. 4F–M.

**Fig. 5:**
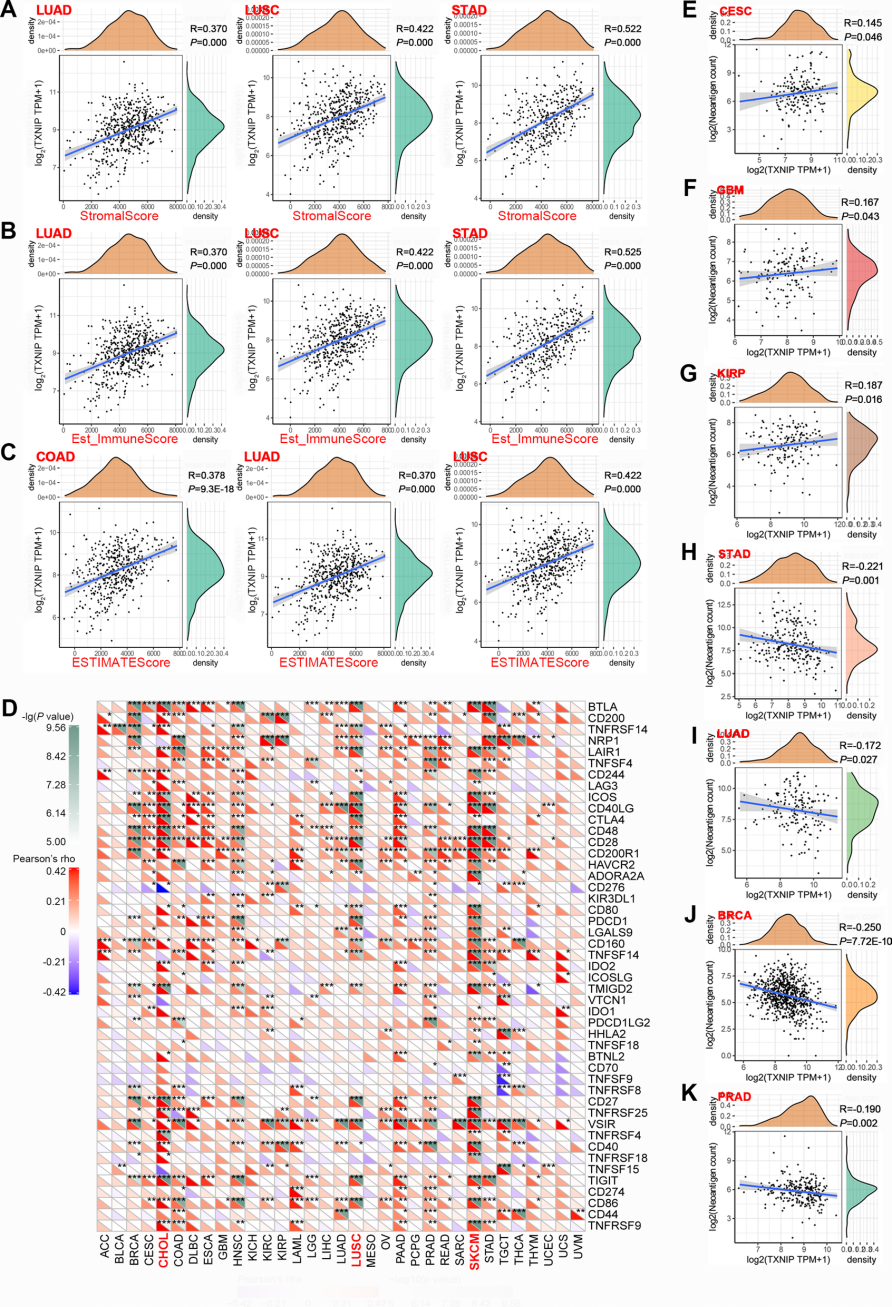
Correlation of TXNIP expression with immune and matrix scores, immune checkpoints, and neoantigens in different tumors of TCGA. **A**–**C** Correlation of *TXNIP* expression with the immune and matrix scores in LUAD, LUSC, COAD, and STAD. **D** Correlation of *TXNIP* expression with 47 transcripts related to immune checkpoints. **E**–**K** Correlation of *TXNIP* expression with the numbers of neoantigen

To further assess the stromal content (StromalScore), overall immune infiltration (Est_ImmuneScore), and the combined score (ESTIMATEScore) of the tumor tissues, the ESTIMATE algorithm was employed [31, 32]. As shown in Fig. 5A–C, the *TXNIP* expression was significantly positively correlated with the immune and matrix scores in the tumors of LUAD, LUSC, and STAD (all *P* < 0.001). Mounting preclinical and clinical evidence discloses that immune checkpoint blockade therapy is emerging as one of the most promising strategy in oncology [18]. Next, in order to identify the potential therapeutic targets and further interpret the immune microenvironment, we analyzed the expression of a set of immune checkpoints in 33 types of studied tumors. Many types of tumors, especially CHOL, LUSC, and SKCM (Fig. 5D), highly express multiple immune checkpoint molecules, indicating that combinatorial treatment with different checkpoint inhibitors may be required for optimal tumor control. Increasing evidence suggests that tumor neoantigen plays a pivotal role in antitumor immune response and cancer immunotherapies [19]. Our data showed that the numbers of neoantigen in tumors like CESC (Fig. 5E), KIRP (Fig. 5G), and glioblastoma multiforme (GBM, Fig. 5F) was significantly positively associated with the expression of *TXNIP* (all *P* < 0.05). However, tumors such as STAD (Fig. 5H), LUAD (Fig. 5I), BRCA (Fig. 5J), and PRAD (Fig. 5K) displayed a negative association between the numbers of neoantigen and *TXNIP* expression (all *P* < 0.05).

### Enrichment analysis of *TXNIP*-related partners

**Fig. 6:**
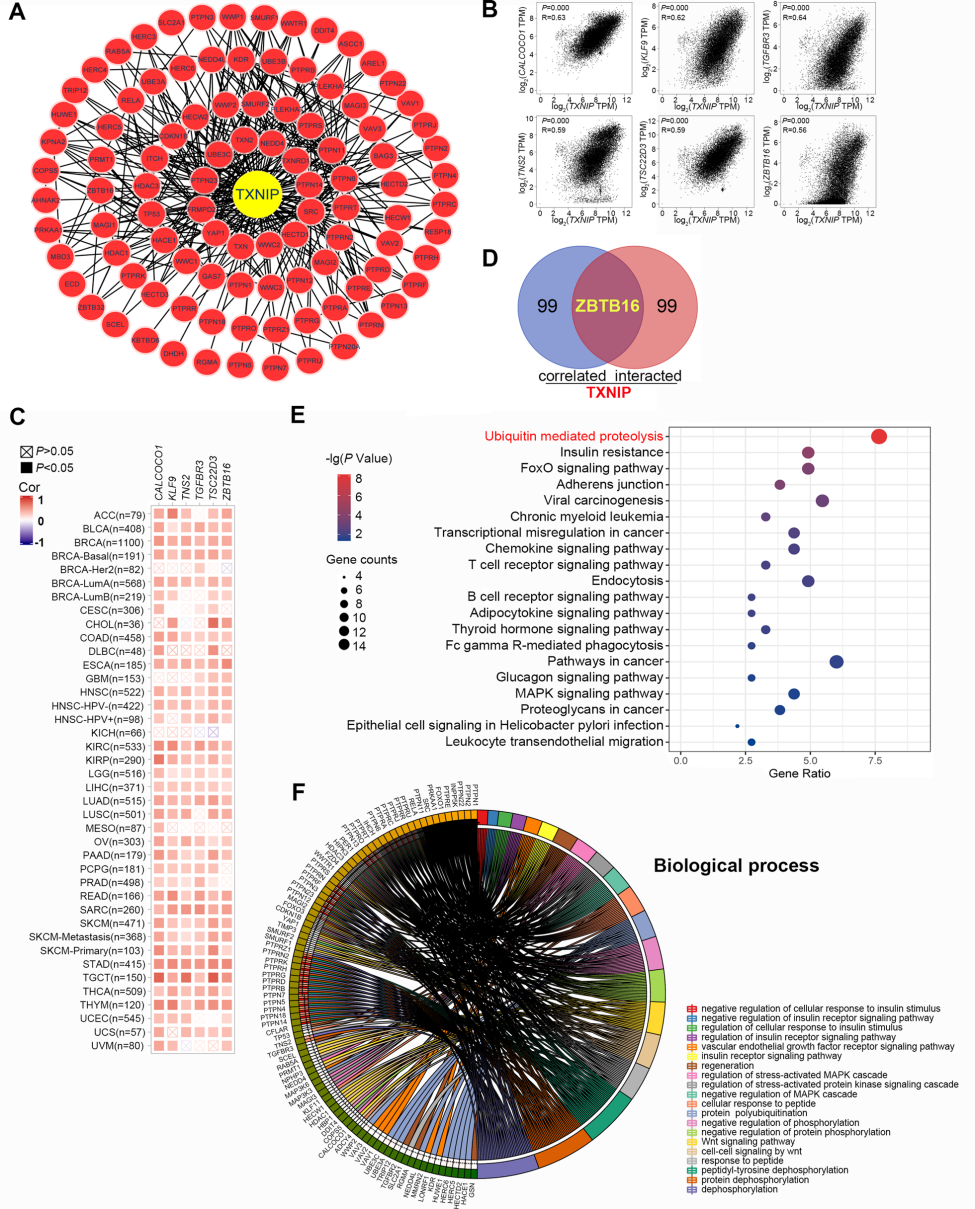
TXNIP-related gene enrichment analysis. **A** Protein–protein interaction network of TXNIP and its experimentally verified binding proteins. **B** The corresponding scatterplot of *TXNIP* and its correlated genes in TCGA projects, including *CALCOCO1*, *KLF9*, *TGFBR3*, *TNS2*, *TSC22D3* and *ZBTB16*. **C** The corresponding heatmap data of *TXNIP* expression and interacted genes. **D** Venn Diagram of the TXNIP-binding and correlated genes. **E** KEGG pathway analysis of TXNIP-binding and interacted genes. **F** Circular plot of the biological processes enriched of *TXNIP*-binding and interacted genes

To further conduct the pathway enrichment analyses concerning the TXNIP-correlated proteins or genes, a group of public tools were applied, of which includes the STRING tool and the GEPIA2 tool. We first collected 100 binding proteins targeting TXNIP using the STRING tool, which were obtained in the setting of “active interaction sources: Experiments”. And the protein–protein interaction (PPI) network of these molecule is shown in Fig. 6A. Next, the top 100 *TXNIP* expression-related genes was extracted from the TCGA data using the GEPIA2 tool. As displayed in Fig. 6B, the expression level of *TXNIP* was significantly correlated with that of *CALCOCO1* (calcium binding and coiled-coil domain 1, R = 0.63), *KLF9* (Kruppel like factor 9, R = 0.62), *TGFBR3* (transforming growth factor beta receptor 3, R = 0.64), *TNS2* (tensin 2, R = 0.59), *TSC22D3* (TSC22 domain family member 3, R = 0.59) and *ZBTB16* (zinc finger and BTB domain-containing protein 16, R = 0.56) genes (all *P* = 0.000). The positive correlations between *TXNIP* and one or more of the above six genes was also observed in most cancer types according to the heatmap data (Fig. 6C). A specific molecule, *ZBTB16*, was obtained from the intersection analysis of the above two groups (Fig. 6D).

The KEGG and GO enrichment analyses were successively performed based on the two datasets. Results of the KEGG enrichment analysis suggested that “ubiquitin mediated proteolysis” ranked first among those enriched pathways, suggesting that it may be related to the role of *TXNIP* in tumor pathogenesis (Fig. 6E). Most of the aforementioned genes were closely linked to the cellular biological process of PTMs, such as protein dephosphorylation or phosphorylation, polyubiquitination, phosphoric ester hydrolase activity, protein phosphorylated amino acid binding, receptor tyrosine kinase binding, according to our GO enrichment analysis data (Fig. 6F). Meanwhile, pathways including cell–cell signaling by Wnt, stress-activated protein kinase signaling cascade, insulin receptor signaling pathway, and vascular endothelial growth factor receptor signaling pathway were related to those genes (Additional file 7: Fig. S7).

## Discussion

The multifunctional TXNIP protein has been reported to participate in an ocean of cellular biological processes, including the cell cycle, apoptotic process, cell division and gene transcription regulation [3–7]. In the current study, the alignment of TXNIP protein sequences across species reveals strong structure element conservation. Emerging evidences have proclaimed the functional link between *TXNIP* and tumors. The specific role of *TXNIP* in the pathogenesis of different tumors remains much to be explored. Unfortunately, we failed to retrieve any pan-cancer analysis of *TXNIP*. Hence, we comprehensively analyzed the *TXNIP* gene in various tumors via the public databases from the perspective of gene expression, DNA methylation, genetic alteration, protein ubiquitination or phosphorylation, and related pathways.

In most tumors of our study, the expression of *TXNIP* was lower than that in matched normal tissues. Nevertheless, for the gene of *TXNIP*, data on the survival prognosis analysis conducted between different databases suggested inconsistent conclusions for different tumors. For example, high expression of *TXNIP* was associated with a more favorable prognosis for patients with KIRC in GEPIA2, while there was no significant effect on prognosis in Kaplan–Meier-plotter and the PrognoScan database. Similarly, elevated *TXNIP* and a good prognosis of breast cancer and lung cancer were only correlated in the data sets of Kaplan–Meier-plotter and PrognoScan, but not of the GEPIA2. However, in these databases, we found consistent results regarding prognosis in most tumor types (breast cancer, lung cancer, liver cancer, bladder cancer, and UVM). STAD and CRC were exceptions where low levels of *TXNIP* expression showed a better prognosis. (Fig. 2A, O, Additional file 2: Fig. S2T). The discrepancies in the prognostic role of *TXNIP* in various tumors in different databases may be a reflection in data collection and processing mechanisms and the underlying mechanisms pertinent to different biological properties. It’s worth noting that the prognostic values of *TXNIP* in gastric cancer were controversial (Fig. 2N, O). Interestingly, when we confined the data of gastric cancer patients with stage 2 to 4 disease, patients with lymph node metastasis, and patients treated with surgery alone, a correlation between decreased *TXNIP* and good survival outcomes was observed (Additional file 8: Table S2). Thus, necessary clinical features should be fully taken into consideration as well. Together these findings powerfully draw a systematic prognostic landscape for *TXNIP* and demonstrate that *TXNIP* is a prognostic biomarker for breast, lung, liver, and bladder cancer.

MSI has been demonstrated to be associated with poor differentiation, proximal localization and chemotherapy efficacy of tumor [23, 24]. In this study, we first presented evidence of the potential correlation between *TXNIP* expression and MSI/TMB across all TCGA tumors. An MMR deficiency may result In MSI and carcinogenesis [13, 33]. Consistently, our findings showed that five genes related to the MMR of DNA were positively linked to *TXNIP* expression in many types of tumor (Fig. 3G, all *P* < 0.01). DNA methylation, catalyzed by the DNMT family, is one of the most important epigenetic modifications, and its role in carcinogenesis has become a topic of considerable interest in recent years [25]. Moreover, MSI has been reported to frequently occur in the hypermethylated cases [24]. For KIRC, KIRP, PRAD and PCPG patients of TCGA, we observed a close correlation between up-regulated expression of *TXNIP* and down-regulated DNMTs using the Sangerbox platform (Fig. 3G, all *P* < 0.05). A recent publication notes that *TXNIP* promoter methylation may mediate the carcinogenesis of human papillomavirus (HPV)-induced cervical cancer [34]. All in all, these results congruously reveal the alteration characteristics of anti-oncogene *TXNIP* in different tumors and its potential association with tumorigenesis.

We applied multiple immune deconvolution approaches to obtain positive correlations between *TXNIP* expression and the immune infiltration levels of diverse TIICs in various types of cancers (Fig. 4A). We found that resting state macrophages (M0) displayed negative correlations with *TXNIP* in some specific TCGA tumors, but polarized macrophages, especially M2 macrophages, showed significant positive correlations. Given the fact that macrophage polarization status are intimately linked to their biological functions, our findings suggest a possible activating effect of *TXNIP* in the polarization of TAMs. Furthermore, BRCA, LUAD and KIRC were recognised as the three tumors with the most significant correlation both with oncologic outcomes and infiltrating immune cells score. CAFs were reported to regulate the function of various TIICs in TME [30], and our findings first suggested the association of *TXNIP* expression and the estimated infiltration value of CAFs in tumors of BRCA, BRCA-Basal, COAD, LIHC, LUSC, READ, STAD, and TGCT (Fig. 4E–M, all *P* < 0.05). In addition, based on the analyses of the immune and matrix scores, immune checkpoints, and tumor neoantigen, we obtained the specific immunologic landscape of *TXNIP* in different tumors. Altogether, our study provides insights in understanding the potential role of *TXNIP* in tumor immune microenvironment and its possible application in tumor immunotherapy.

*ZBTB16* (Zinc finger and BTB domain-containing protein 16), a member of the Krueppel (C2H2-type) zinc-finger transcription factor family, represses transcription possibly through recruitment of histone deacetylases to target promoters [35]. This transcriptional repressor may mediate the ubiquitination and subsequent proteasomal degradation of target proteins [36]. Strikingly, the intersection analysis of the *TXNIP* expression-correlated genes indicated that the expression of *ZBTB16* was closely related to that of *TXNIP* (Fig. 6D). Additionally, our protein modification sites analysis predicted that *TXNIP* has multiple modification sites, especially sites for ubiquitination and phosphorylation (Fig. 3H). To further conduct the KEGG and GO enrichment analyses, we integrated the information of *TXNIP* expression-correlated genes and *TXNIP*-binding components across all tumors, and identified the potential impact of “ubiquitin mediated proteolysis”, “protein dephosphorylation or phosphorylation”, “polyubiquitination”, “MAPK signaling pathway”, and “Wnt signaling pathway” in cancer pathogenesis.

## Conclusion

In summary, our first pan-cancer analysis of *TXNIP* indicated that there were statistical correlations between *TXNIP* expression and multiple molecular characteristics across different kinds of tumors, including clinical prognosis, TMB/MSI, MMR genes, DNA methylation, immune cell infiltration, protein ubiquitination and phosphorylation. These findings contribute to a more comprehensive understanding of the role of *TXNIP* in tumorigenesis. However, the limitation of the study is the lack of some validating data. It may also be necessary to extend the follow-up period for prognostic analysis, so as to obtain more accurate and complete information. All in all, *TXNIP* plays an important role in tumorigenesis and tumor immunity, and may be a potential prognostic and therapeutic biomarker.

## Supplementary Information

**Additional file 1: Figure S1.** General characteristics of TXNIP.

**Additional file 2: Figure S2.** Survival curves comparing the high and low expression of TXNIP in different tumors in the Kaplan–Meier plotter (**A**–**H**) and PrognoScan databases (**I**–**T**).

**Additional file 3: Figure S3.** Correlation of TXNIP expression with tumor-infiltrating immune cells in various types of cancers via the TIMER2 database.

**Additional file 4.** Correlation of TXNIP expression with tumor-infiltrating immune cells in MESO, LUSC, THYM, THCA, TGCT, SATD, SKCM and SARC via the TIMER2 database.

**Additional file 5.** Correlation of TXNIP expression with tumor-infiltrating immune cells in KIRP, LIHC, LGG, DLBC, COAD, CHOL, CESC and KICH via the TIMER2 database.

**Additional file 6.** Correlation of TXNIP expression with tumor-infiltrating immune cells in HNSC, GBM, ESCA, ACC and BLCA via the TIMER2 database.

**Additional file 7: Figure S4.** TXNIP-related gene enrichment analysis. Circular plot of the cellular components (A) and molecular functions (B) enriched for the interest genes.

**Additional file 8.** Supplementary data.

## Data Availability

Publicly available data sets were analyzed in this study. All of the data in this article were used the TCGA datasets (https://www.cancer.gov/about-nci/organiza-tion/ccg/research/structural-genomics/tcga), GTEx datasets (https://www.genome.go-v/Funded-Programs-Projects/Genotype-Tissue-Expression-Project) and GEO databases (https://www.ncbi.nlm.nih.gov/geo/).
