# Supplementary figures and images for: A pan-cancer analysis of thioredoxin-interacting protein as an immunological and prognostic biomarker
Source: Cancer Cell Int. 2022 Jul 17;22:230. doi: 10.1186/s12935-022-02639-2 (PMC9288722; doi:10.1186/s12935-022-02639-2)

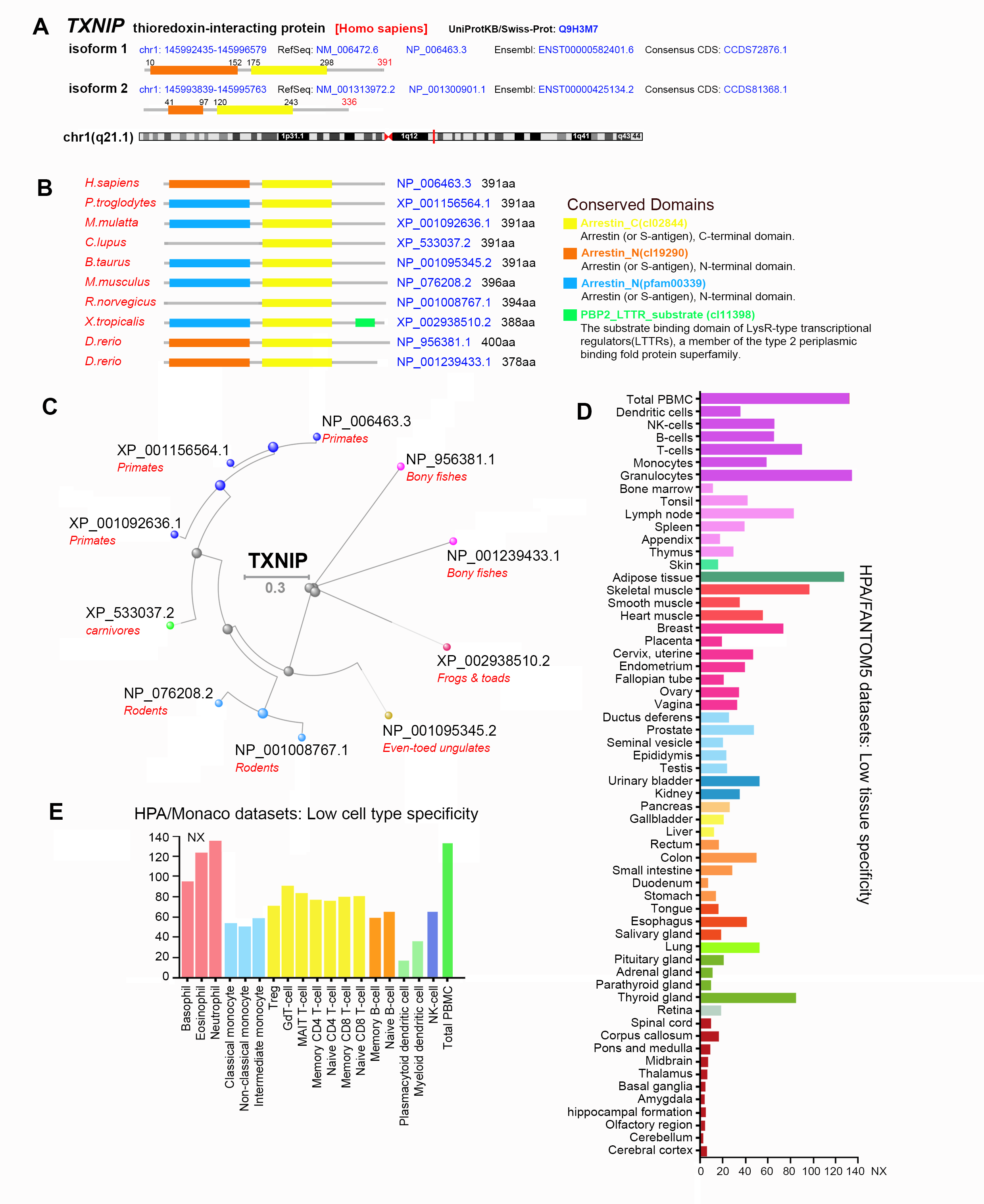

Supplement: Supplementary file 1 — Additional file 1: Figure S1. General characteristics of TXNIP. [file 12935_2022_2639_MOESM1_ESM.tif]

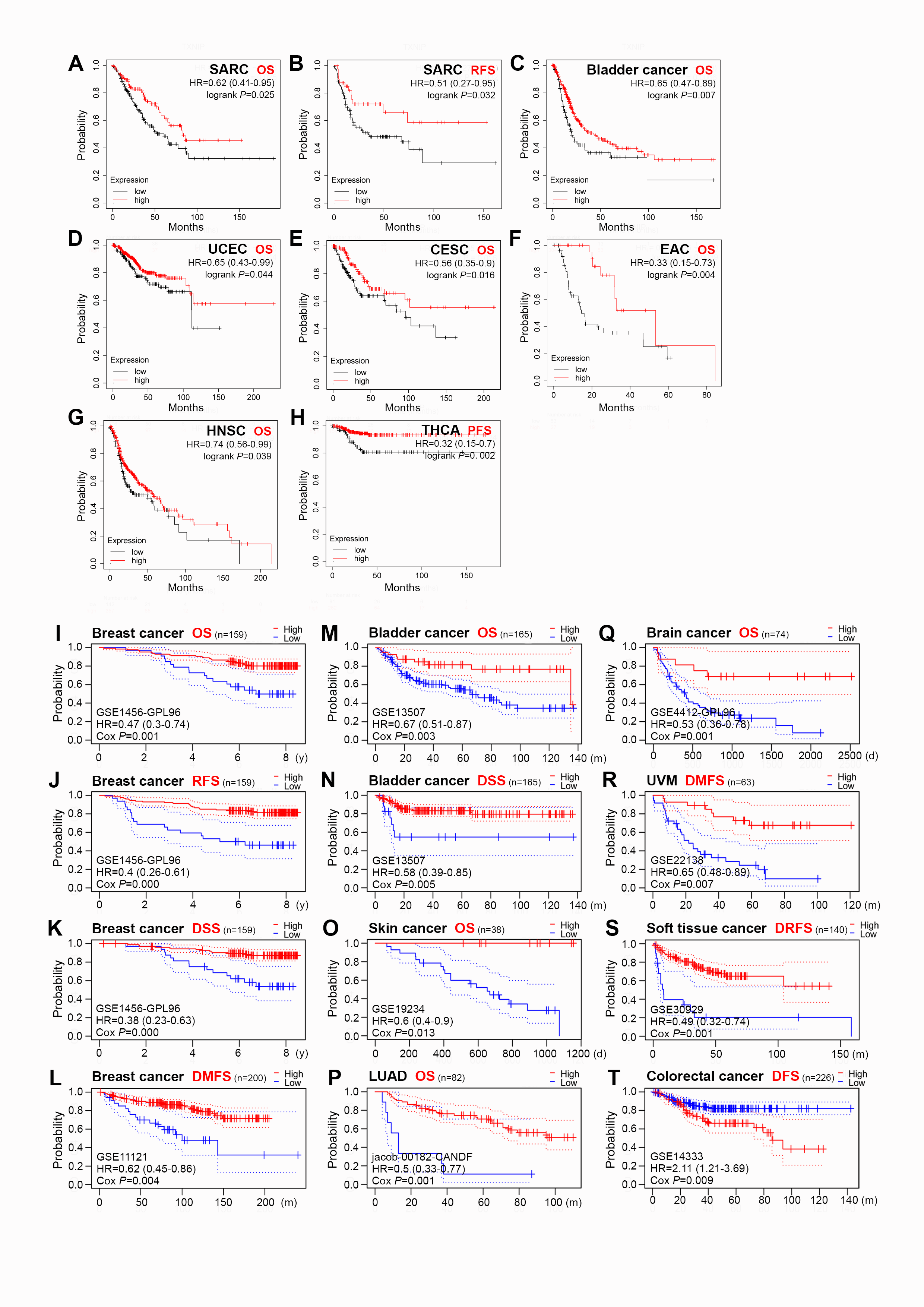

Supplement: Supplementary file 2 — Additional file 2: Figure S2. Survival curves comparing the high and low expression of TXNIP in different tumors in the Kaplan–Meier plotter (A–H) and PrognoScan databases (I–T). [file 12935_2022_2639_MOESM2_ESM.tif]

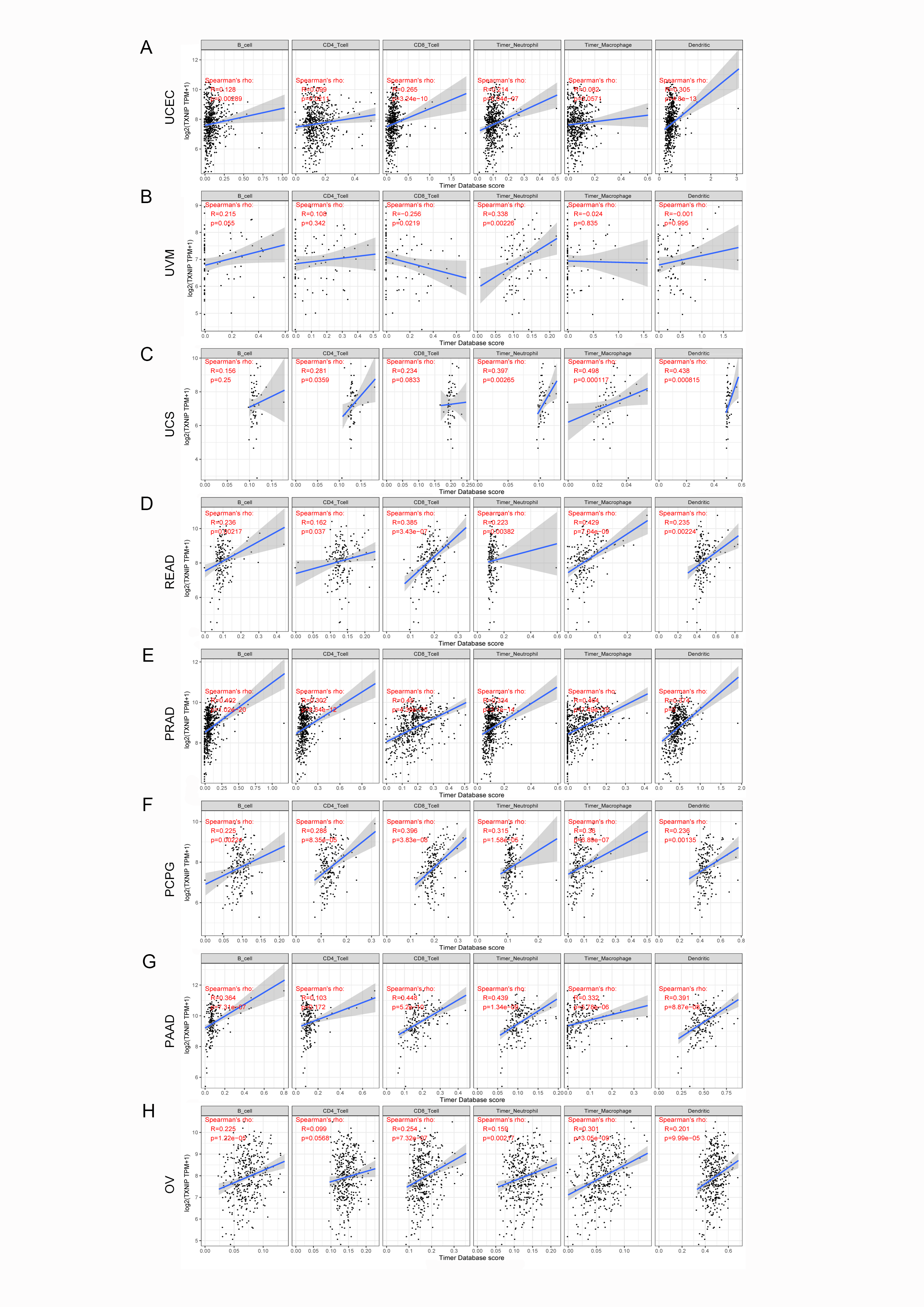

Supplement: Supplementary file 3 — Additional file 3: Figure S3. Correlation of TXNIP expression with tumor-infiltrating immune cells in various types of cancers via the TIMER2 database. [file 12935_2022_2639_MOESM3_ESM.tif]

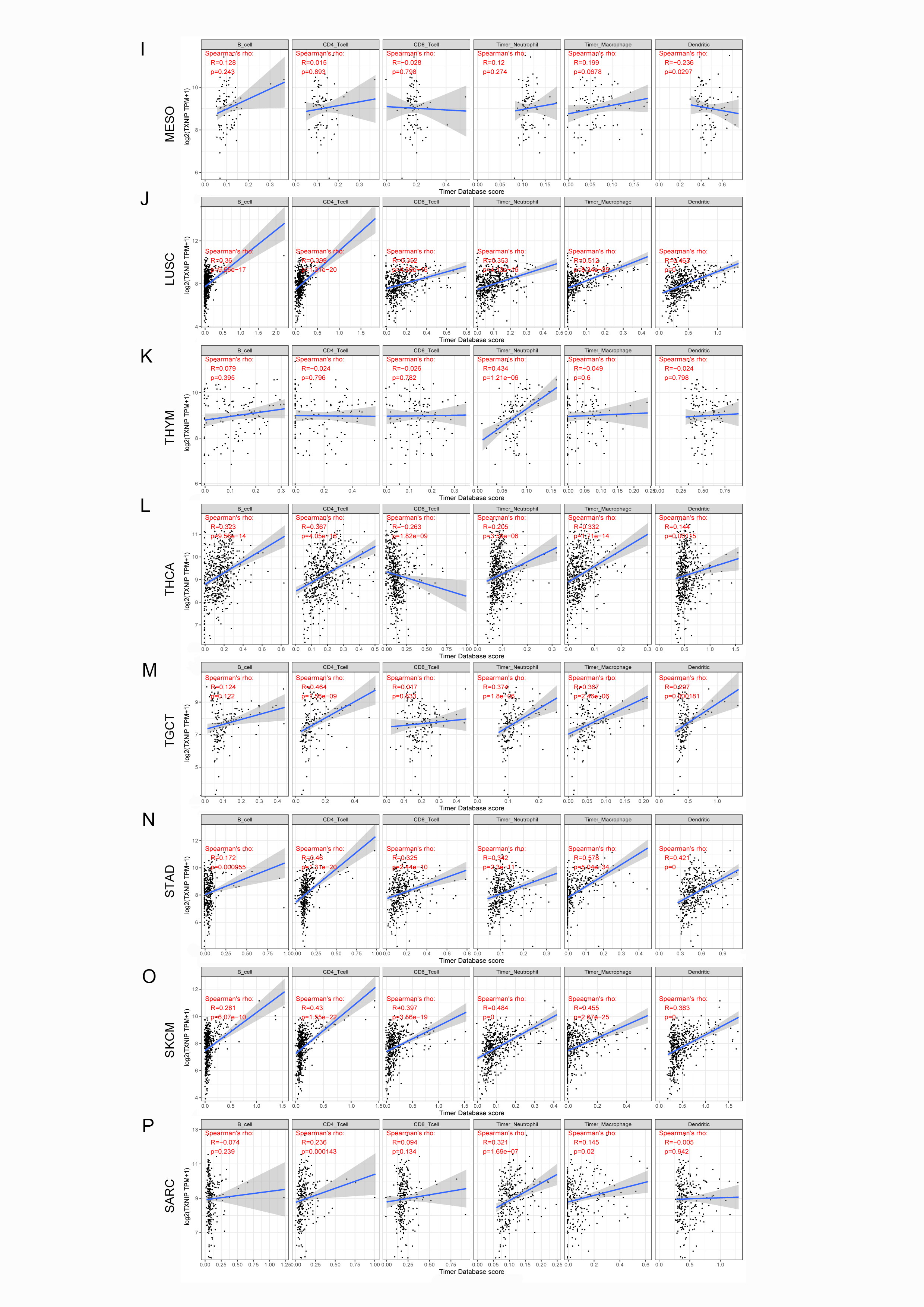

Supplement: Supplementary file 4 — Additional file 4. Correlation of TXNIP expression with tumor-infiltrating immune cells in MESO, LUSC, THYM, THCA, TGCT, SATD, SKCM and SARC via the TIMER2 database. [file 12935_2022_2639_MOESM4_ESM.tif]

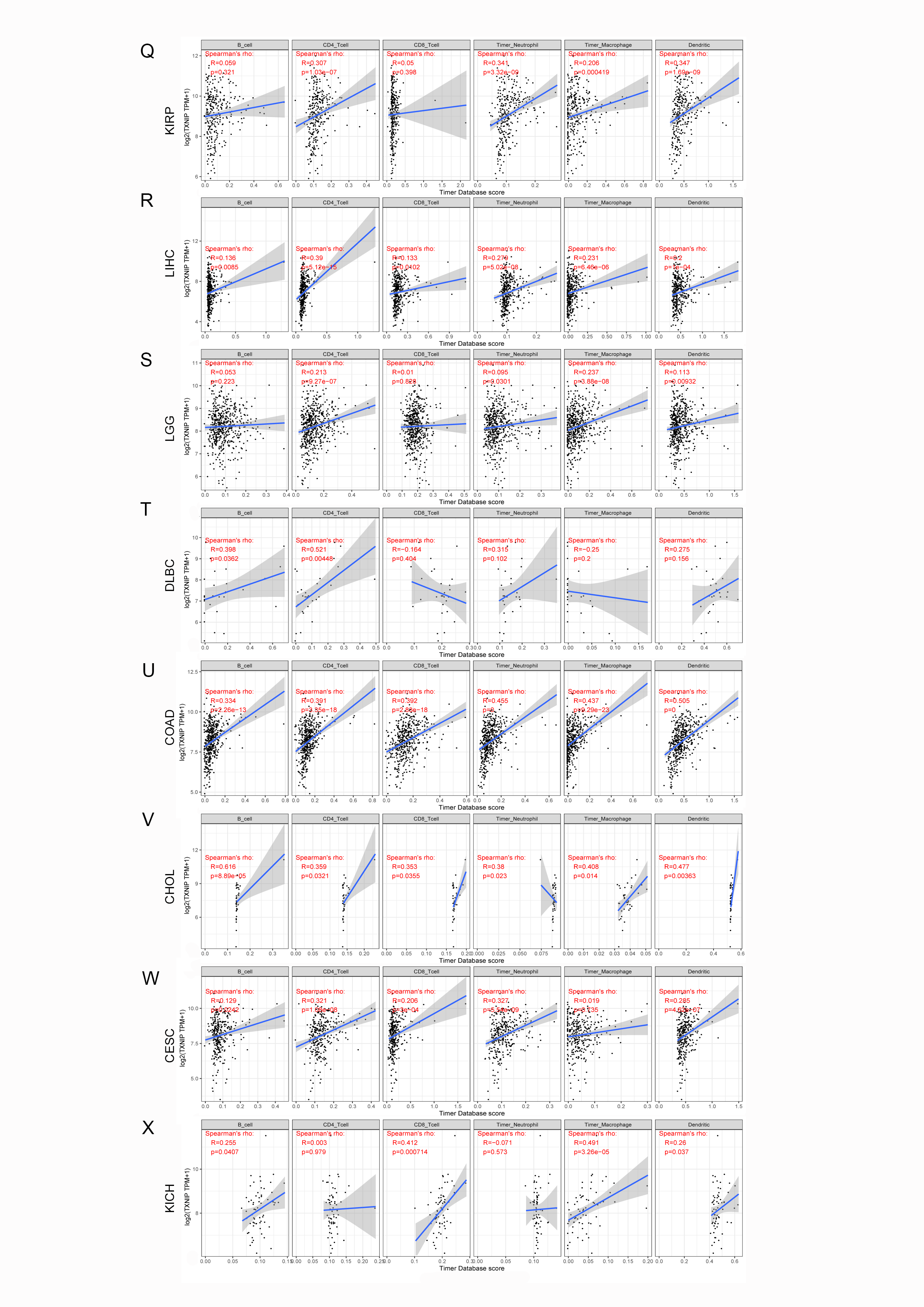

Supplement: Supplementary file 5 — Additional file 5. Correlation of TXNIP expression with tumor-infiltrating immune cells in KIRP, LIHC, LGG, DLBC, COAD, CHOL, CESC and KICH via the TIMER2 database. [file 12935_2022_2639_MOESM5_ESM.tif]

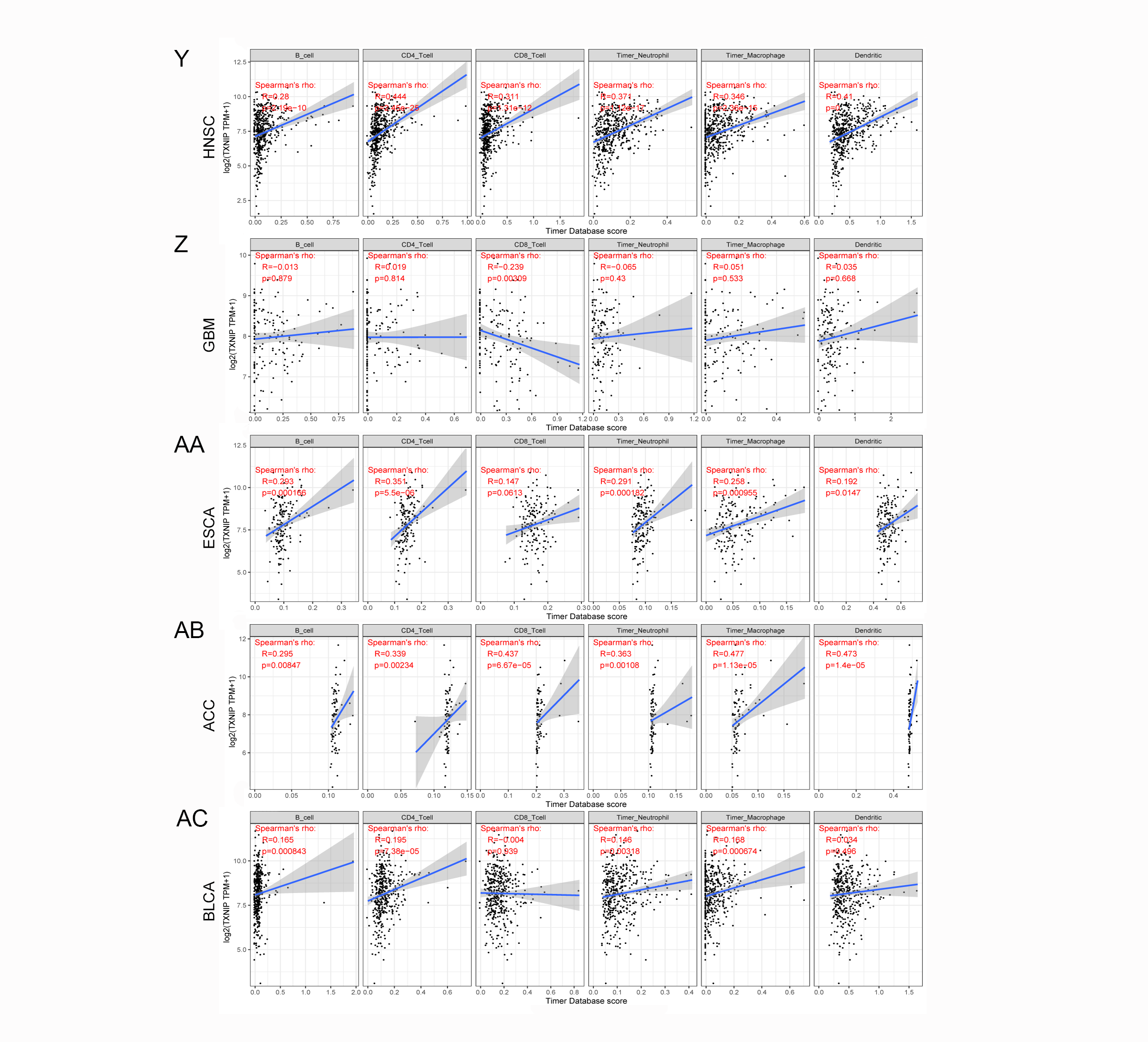

Supplement: Supplementary file 6 — Additional file 6. Correlation of TXNIP expression with tumor-infiltrating immune cells in HNSC, GBM, ESCA, ACC and BLCA via the TIMER2 database. [file 12935_2022_2639_MOESM6_ESM.tif]

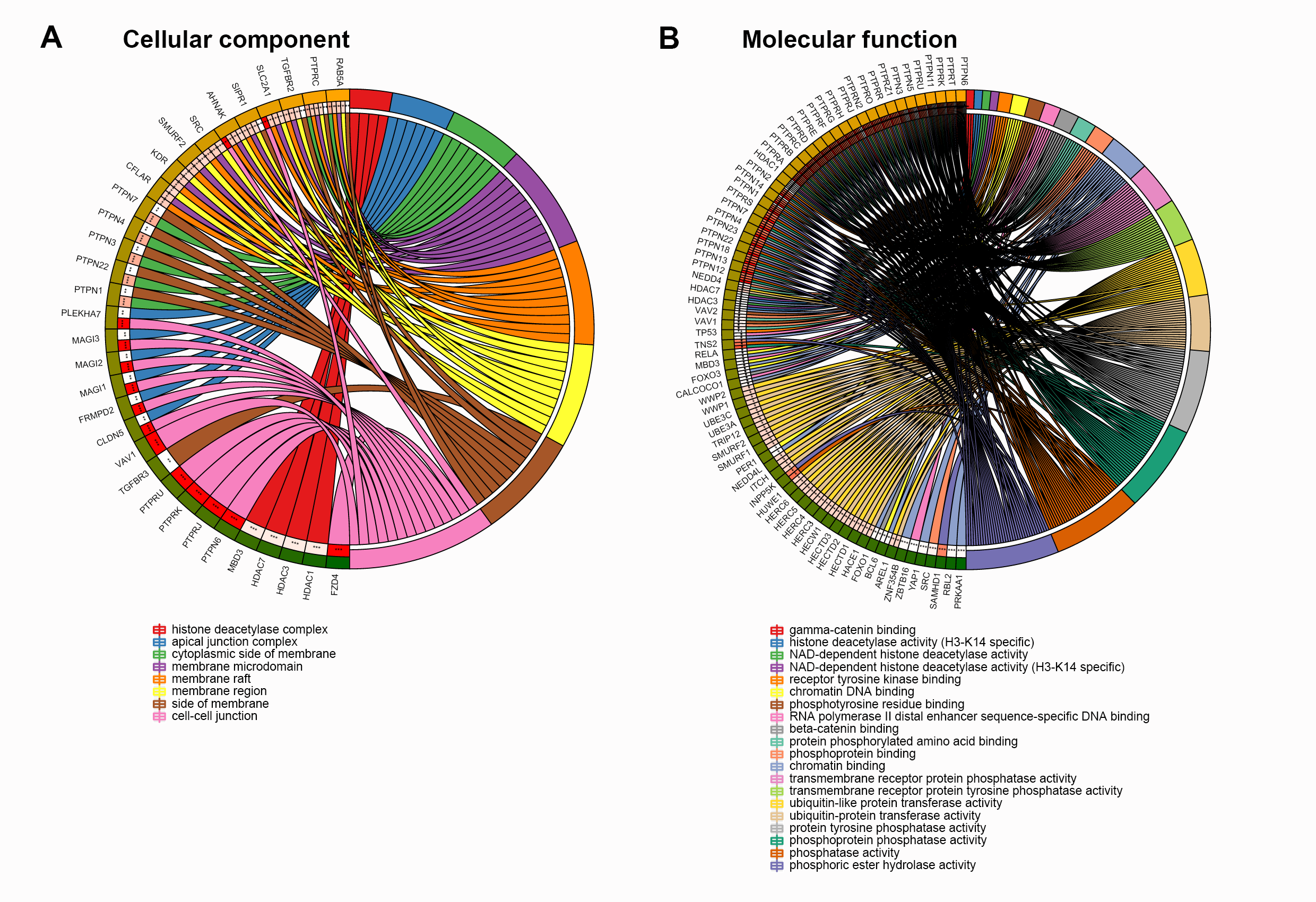

Supplement: Supplementary file 7 — Additional file 7: Figure S4. TXNIP-related gene enrichment analysis. Circular plot of the cellular components (A) and molecular functions (B) enriched for the interest genes. [file 12935_2022_2639_MOESM7_ESM.tif]
