# Supplementary material for: A pan-cancer analysis of thioredoxin-interacting protein as an immunological and prognostic biomarker
Source: Cancer Cell Int. 2022 Jul 17;22:230. doi: 10.1186/s12935-022-02639-2 (PMC9288722; doi:10.1186/s12935-022-02639-2)
Supplement: Supplementary file 8 — Additional file 8. Supplementary data. [file 12935_2022_2639_MOESM8_ESM.docx]

**Supplementary data**

**A pan-cancer analysis of** **thioredoxin-interacting protein as an immunological and prognostic biomarker**

# 1. Supplementary materials and methods

## 1.1 Gene expression analysis of HPA and FANTOM5

The TXNIP expression pattern in different cells and normal human tissues was analyzed via the online Human Protein Atlas (HPA) database (https://www.proteinatlas.org/humanproteome/pathology)^1^and Function Annotation of the Mammalian Genome 5 (FANTOM5) datasets (<https://fantom.gsc.riken.jp/5/>).

## 1.2 Survival prognosis analysis in PrognoScan database

PrognoScan database (http://www.prognoscan.org/) provides a powerful platform for evaluating the biological relationship between gene expression and patient prognosis across a large collection of publicly available cancer microarray datasets with clinical annotation^2^. The associations between TXNIP expression levels and survival in different cancers was obtained from the tool. Survival curves for high and low expression groups dichotomized at the optimal cutpoint are plotted. The threshold was adjusted to a Cox *P*-value < 0.05.

## 1.3 Immune infiltration analysis

The TIMER2 web server, a powerful tool for the systematic analysis of immune infiltrates across diverse cancer types from gene expression profiles, was used to visualize the correlation of TXNIP expression with immune infiltration level across all TCGA tumors. The TXNIP expression in different cancers and its correlation with the abundances of eight tumor-infiltrating immune cells (TIICs), i.e. B cells, CD4^+^ T cells, CD8^+^ T cells, neutrophils, dendritic cells, macrophages, monocytes, and regulatory T lymphocytes (Tregs), together with cancer-associated fibroblasts (CAFs) was explored by the corresponding gene modules. The TIMER, EPIC, quanTIseq, xCell, MCP-counter, CIBERSORT, CIBERSORT-ABS, and TIDE algorithms were applied for immune infiltration estimations. The *P*-values and partial correlation (cor) values were obtained via the purity-adjusted Spearman’s rank correlation test.

# 2. Supplementary figure legends

## Fig. S1. General characteristics of TXNIP

**(A)** Genomic location of human TXNIP. **(B)** Conserved domains of TXNIP protein among different species. TXNIP is highly conserved among species, and commonly consists of the Arrestin_C (cl02844) domain. **(C)** Phylogenetic tree of TXNIP in different species was obtained via a constraint-based multiple alignment tool of NCBI. Expression level of the TXNIP gene in different tissues **(D)** and cells **(E)** in the normal physiological state.

## Fig. S2. Survival curves comparing the high and low expression of TXNIP in different tumors in the Kaplan-Meier plotter (A–H) and PrognoScan databases (I–T)

Survival curves of OS, RFS and PFS, in SARC **(A, B)**, bladder cancer **(C)**, UCEC **(D)**, CESC **(E)**, EAC **(F)**, HNSC **(G)**, and THCA **(H)**. **(I–L)** Survival curves of OS, RFS, DSS, and DMFS in two breast cancer cohorts. **(M, N)** Survival curves of OS and DSS in the bladder cancer cohort. **(O–Q)** Survival curves of OS in the LUAD, skin and brain cancer cohorts. **(R)** Survival curves of DMFS in the UVM cohort. **(S)** Survival curves of DRFS in a soft tissue cancer cohort. **(T)** Survival curves of DFS in the colorectal cancer cohort.

## Fig. S3. Correlation of TXNIP expression with tumor-infiltrating immune cells in UCEC, UVM, UCS, READ, PRAD, PCPG, PAAD and OV via the TIMER2 database

## Fig. S4. Correlation of TXNIP expression with tumor-infiltrating immune cells in MESO, LUSC, THYM, THCA, TGCT, SATD, SKCM and SARC via the TIMER2 database

## Fig. S5. Correlation of TXNIP expression with tumor-infiltrating immune cells in KIRP, LIHC, LGG, DLBC, COAD, CHOL, CESC and KICH via the TIMER2 database

## Fig. S6. Correlation of TXNIP expression with tumor-infiltrating immune cells in HNSC, GBM, ESCA, ACC and BLCA via the TIMER2 database

## Fig. S7. TXNIP-related gene enrichment analysis. Circular plot of the cellular components (A) and molecular functions (B) enriched for the interest genes.

# 3.Supplementary table

**Supplementary Table 1.** Human TXNIP expression in cancers vs. normal tissue in Oncomine database

| **Cancer** | **Cancer type** | ***P*-value** | **Fold change** | **Rank (%)** | **Sample** | **Reporter** |
| --- | --- | --- | --- | --- | --- | --- |
| Bladder  Breast  Cervical  Esophageal  Gastric  Head and Neck  Leukemia  Liver  Lung  Brain and Central nervous system  Ovarian  Sarcoma  Lymphoma  Melanoma  Other | Superficial Bladder Cancer  Infiltrating Bladder Urothelial Carcinoma  Mucinous Breast Carcinoma  Invasive Ductal and Invasive Lobular Breast Carcinoma  Ductal Breast Carcinoma  Invasive Lobular Breast Carcinoma  Mixed Lobular and Ductal Breast Carcinoma  Invasive Ductal Breast Carcinoma  Invasive Ductal and Lobular Carcinoma  Medullary Breast Carcinoma  Cervical Squamous Intraepithelial Neoplasia Epithelia  Cervical Squamous Cell Carcinoma  Cervical Non-Keratinizing Squamous Cell Carcinoma  Cervical Squamous Cell Carcinoma  Esophageal Squamous Cell Carcinoma  Gastric Cancer  Gastric Intestinal Type Adenocarcinoma  Gastric Adenocarcinoma  Mucinous Gastric Adenocarcinoma  Thyroid Gland Papillary Carcinoma  Tall Cell Variant Thyroid Gland Papillary Carcinoma  Oral Cavity Carcinoma  Chronic Lymphocytic Leukemia  Chronic Lymphocytic Leukemia  Chronic Lymphocytic Leukemia  Chronic Lymphocytic Leukemia  B-Cell Acute Lymphoblastic Leukemia  Hepatocellular Carcinoma  Hepatocellular Adenoma  Hepatocellular Carcinoma  Hepatocellular Carcinoma  Hepatocellular Carcinoma  Lung Adenocarcinoma  Lung Mucinous Adenocarcinoma  Acinar Lung Adenocarcinoma  Non-Mucinous Bronchioloalveolar Carcinoma  Lung Adenocarcinoma  Squamous Cell Lung Carcinoma, Basaloid Variant  Papillary Lung Adenocarcinoma  Micropapillary Lung Adenocarcinoma  Lung Adenocarcinoma  Diffuse Astrocytoma  Oligodendroglioma  Anaplastic Astrocytoma  Glioblastoma  Brain Glioblastoma  Glioblastoma  Glioblastoma  Oligodendroglioma  Ovarian Serous Cystadenocarcinoma  Sarcoma  Uterine Corpus Leiomyosarcoma  Angioimmunoblastic T-Cell Lymphoma  Diffuse Large B-Cell Lymphoma  Cutaneous Melanoma  Endometrial Endometrioid Adenocarcinoma  Endometrial Serous Adenocarcinoma  Endometrial Mixed Adenocarcinoma | 1.30e-06  4.26e-06  0.004  0.021  0.030  3.57e-18  0.019  2.93e-94  0.001  0.049  0.002  3.06e-15  0.003  3.95-06  3.45e-05  5.75e-06  1.21e-04  1.40e-12  0.016  9.01e-07  0.013  0.008  2.14e-05  3.86e-05  0.001  0.002  0.010  8.25e-07  5.04e-04  6.12e-21  1.68e-16  7.04e-06  1.33e-17  0.025  0.015  0.004  8.57e-34  0.002  0.006  0.040  7.49e-11  1.55e-07  1.59e-15  3.07e-08  4.56e-12  2.01e-11  0.003  3.81e-06  0.021  1.78e-129  2.95e-06  0.023  4.93e-05  0.035  5.03e-10  4.27e-30  4.88e-09  0.009 | 3.183  1.123  1.243  1.424  1.160  1.251  1.183  1.219  1.201  1.364  2.048  1.155  1.144  1.076  1.098  1.064  1.048  1.068  1.081  1.020  1.027  1.838  4.184  2.529  5.048  3.977  1.378  2.692  1.917  1.282  1.087  1.085  1.279  1.138  1.127  1.158  1.169  1.359  1.219  1.128  1.123  2.607  3.835  3.938  2.781  2.613  2.858  1.998  1.462  1.243  1.186  1.180  2.107  1.054  1.162  1.160  1.179  1.102 | 7%  6%  5%  5%  5%  5%  5%  6%  7%  8%  6%  5%  9%  8%  10%  2%  9%  8%  10%  6%  6%  9%  5%  5%  7%  10%  7%  7%  2%  2%  4%  5%  2%  3%  3%  5%  5%  7%  7%  10%  6%  1%  1%  2%  5%  3%  8%  5%  10%  2%  2%  5%  9%  8%  7%  1%  4%  5% | 776  950  883  900  901  902  906  1000  1231  1431  664  844  1596  1400  1863  289  1509  1432  1781  956  1012  1703  122  633  164  400  855  804  163  373  596  863  263  418  492  907  939  1135  1249  1765  1022  12  63  326  956  278  952  686  1415  348  277  866  1682  1336  1204  136  670  766 | 201008_s_at  01-144151901  01-144151901  01-144151901  01-144151901  01-144151901  01-144151901  01-144151901  01-144151901  01-144151901  201009_s_at  01-144151901  01-144151901  01-144151901  01-144151901  01-144151901  01-144151901  01-144151901  01-144151901  01-144151901  01-144151901  201008_s_at  IMAGE:488488  IMAGE:488488  AA044633(4)  AA044633(2)  201008_s_at  201010_s_at  IMAGE:1554439  01-144151901  01-144151901  01-144151901  01-144151901  01-144151901  01-144151901  01-144151901  01-144151901  01-144151901  01-144151901  01-144151901  01-144151901  201010_s_at  201008_s_at  201008_s_at  201008_s_at  201009_s_at  201009_s_at  IMAGE:1554367  IMAGE:1554439  01-144151901  01-144151901  01-144151901  201010_s_at  01-144151901  01-144151901  01-144151901  01-144151901  01-144151901 |

**Supplementary Table 2.** Correlation of *TXNIP* mRNA expression and clinicopathological factors in gastric cancer by Kaplan-Meier plotter database

| **Variables of gastric cancer** | **Overall survival (n=875)** | | | **Post progression survival (n=498)** | | |
| --- | --- | --- | --- | --- | --- | --- |
|  | **Number** | **Hazard ratio** | ***P*-value** | **Number** | **Hazard ratio** | ***P*-value** |
| **Gender**  Female  Male  **Stage**  2  3  4  **Stage T**  2  3  4  **Stage N**  0  1  2  3  1~3  **Stage M**  0  1  **HER2 status**  Negative  Positive  **Lauren classification**  Intestinal  Diffuse  Mixed  **Differentiation**  Poorly  Moderately  Well  **Treatment**  Surgery alone  5 FU based adjuvant  Other adjuvant | 236  544  140  305  148  241  204  38  74  225  121  76  422  444  56  532  343  320  241  32  165  67  32  380  152  76 | 1.29 (0.91-1.83)  1.13 (0.91-1.40)  2.25 (1.24-4.11)  1.41 (1.05-1.89)  1.50 (1.01-2.23)  0.72 (0.47-1.10)  1.41 (1.00-2.00)  2.92 (1.17-7.28)  2.17 (0.94-4.99)  1.80 (1.20-2.72)  1.69 (1.07-2.65)  2.00 (1.15-3.46)  1.68 (1.29-2.19)  1.68 (1.27-2.22)  2.13 (1.17-3.88)  1.25 (1.00-1.56)  0.80 (0.58-1.09)  1.75 (1.27-2.40)  1.62 (1.13-2.32)  0.27 (0.09-0.78)  0.57 (0.37-0.88)  0.74 (0.38-1.45)  0.50 (0.17-1.48)  1.45 (1.08-1.93)  0.58 (0.40-0.84)  1.66 (0.60-4.56) | 0.150  0.270  **0.007**  **0.022**  **0.041**  0.130  0.050  **0.017**  0.062  **0.004**  **0.022**  **0.012**  **0.000**  **0.000**  **0.011**  0.053  0.150  **0.001**  **0.008**  **0.010**  **0.011**  0.380  0.200  **0.012**  **0.003**  0.320 | 149  348  105  142  104  196  150  29  41  169  105  63  337  342  36  334  164  192  176  16  49  24  0  277  135  74 | 1.88 (1.20-2.94)  1.43 (1.09-1.86)  2.38 (1.22-4.63)  1.76 (1.09-2.86)  2.53 (1.34-4.77)  1.57 (0.95-2.60)  1.81 (1.23-2.67)  1.68 (0.67-4.27)  4.36 (1.36-13.90)  2.56 (1.63-4.02)  1.56 (0.95-2.56)  2.51 (1.16-5.46)  1.91 (1.43-2.55)  1.98 (1.47-2.68)  2.92 (1.34-6.35)  1.56 (1.16-2.10)  1.47 (1.03-2.10)  2.01 (1.33-3.05)  2.11 (1.42-3.13)  NA  0.59 (0.30-1.18)  0.60 (0.21-1.67)  NA  1.86 (1.36-2.54)  1.24 (0.82-1.87)  1.77 (0.67-4.67) | **0.005**  **0.008**  **0.008**  **0.020**  **0.003**  0.077  **0.002**  0.270  **0.007**  **2.5e-05**  0.074  **0.016**  **8.0e-06**  **4.8e-06**  **0.005**  **0.003**  **0.033**  **8.0e-04**  **0.000**  NA  0.130  0.320  NA  **7.2e-05**  0.310  0.240 |

Abbreviation: HER2, human epidermalgrowth factor receptor-2.

| **Variables of liver cancer** | **Overall survival (n=364)** | | | **Relapse free survival (n=313)** | | |
| --- | --- | --- | --- | --- | --- | --- |
|  | **Number** | **Hazard ratio** | ***P*-value** | **Number** | **Hazard ratio** | ***P*-value** |
| **Gender**  Female  Male  **Stage**  1+2  3+4  **Grade**  1  2  3  4  **AJCC_T**  1  2  3  4  **Vascular invasion**  None  Micro  Macro  **Alcohol consumption**  None  Yes  **Hepatitis virus**  None  Yes  **Sorafenib treated** | 118  246  253  87  55  174  118  12  180  90  78  13  203  90  16  202  115  167  150  29 | 1.32 (0.69-2.53)  0.32 (0.19-0.55)  0.57 (0.35-0.93)  0.39 (0.19-0.81)  0.45 (0.17-1.20)  0.53 (0.31-0.90)  0.36 (0.17-0.76)  NA  0.55 (0.30-0.99)  0.49 (0.24-1.03)  0.32 (0.13-0.77)  NA  0.59 (0.35-1.00)  0.38 (0.14-1.00)  NA  0.57 (0.36-0.91)  0.38 (0.20-0.72)  0.42 (0.25-0.71)  0.37 (0.18-0.77)  0.13 (0.04-0.51) | 0.410  **1e-05**  **0.022**  **0.009**  0.100  **0.017**  **0.005**  NA  **0.044**  0.053  **0.008**  NA  **0.048**  **0.042**  NA  **0.018**  **0.002**  **0.001**  **0.006**  **6e-04** | 105  208  227  68  45  147  106  11  160  79  65  6  175  81  14  182  98  142  138  22 | 0.55 (0.30-1.01)  0.66 (0.43-1.02)  0.72 (0.47-1.09)  0.49 (0.26-0.92)  0.55 (0.19-1.53)  0.54 (0.33-0.88)  0.71 (0.39-1.28)  NA  0.67 (0.38-1.21)  0.62 (0.32-1.21)  0.53 (0.28-1.01)  NA  0.83 (0.51-1.34)  0.50 (0.25-0.99)  NA  0.63 (0.40-0.98)  1.44 (0.80-2.62)  0.77 (0.46-1.27)  0.69 (0.40-1.18)  1.47 (0.60-3.62) | 0.050  0.061  0.120  **0.023**  0.240  **0.013**  0.250  NA  0.180  0.150  0.050  NA  0.440  **0.043**  NA  **0.041**  0.220  0.300  0.170  0.390 |

**Supplementary Table 3.** Correlation of *TXNIP* mRNA expression and clinicopathological factors in liver cancer by Kaplan-Meier plotter database

## Supplementary Table 4. Correlation of *TXNIP* mRNA expression and clinicopathological factors in lung cancer by Kaplan-Meier plotter database

| **Variables of lung cancer** | **Overall survival (n=1925)** | | | **Post progression survival (n=344)** | | |
| --- | --- | --- | --- | --- | --- | --- |
|  | **Number** | **Hazard ratio** | ***P*-value** | **Number** | **Hazard ratio** | ***P*-value** |
| **Gender**  Female  Male  **Histology**  Adenocarcinoma  Squamous cell carcinoma  **Stage**  1  2  3  **Grade**  I  II  III  **AJCC_M**  0  1  **Smoking history**  Never  Yes  **Chemotherapy**  No  Yes  **Radiotherapy**  No  Yes | 714  1100  719  524  577  244  70  201  310  77  681  10  205  820  310  176  271  70 | 0.65 (0.52-0.83)  0.71 (0.61-0.84)  0.76 (0.60-0.95)  0.67 (0.53-0.85)  0.42 (0.32-0.56)  1.07 (0.74-1.55)  1.30 (0.75-2.24)  0.74 (0.51-1.05)  0.95 (0.69-1.29)  0.67 (0.35-1.30)  0.75 (0.61-0.92)  NA  0.76 (0.43-1.32)  0.71 (0.58-0.88)  0.65 (0.46-0.91)  0.85 (0.56-1.29)  0.77 (0.54-1.10)  0.76 (0.45-1.30) | **0.000**  **2.5e-05**  **0.018**  **0.001**  **5e-10**  0.710  0.340  0.093  0.730  0.230  **0.007**  NA  0.320  **0.001**  **0.011**  0.440  0.150  0.320 | 165  179  125  20  78  58  10  79  89  24  59  0  67  254  97  88  104  57 | 0.52 (0.35-0.76)  0.75 (0.53-1.06)  0.96 (0.60-1.54)  0.85 (0.31-2.37)  0.77 (0.42-1.40)  0.86 (0.45-1.64)  NA  0.59 (0.36-0.97)  0.74 (0.46-1.20)  1.23 (0.45-3.33)  1.66 (0.89-3.08)  NA  1.14 (0.61-2.14)  0.68 (0.51-0.91)  0.45 (0.28-0.72)  0.60 (0.37-0.98)  0.49 (0.31-0.76)  1.13 (0.63-2.02) | **0.001**  0.110  0.880  0.760  0.390  0.640  NA  **0.037**  0.230  0.690  0.110  NA  0.680  **0.009**  **0.001**  **0.038**  **0.001**  0.680 |

| **Variables of breast cancer** | **Overall survival (n=1402)** | | | **Relapse free survival (n=3951)** | | |
| --- | --- | --- | --- | --- | --- | --- |
|  | **Number** | **Hazard ratio** | ***P*-value** | **Number** | **Hazard ratio** | ***P*-value** |
| **Grade**  1  2  3  **Estrogen receptor**  Negative  Positive  **Progesterone receptor**  Negative  Positive  **HER2 status**  Negative  Positive  **Intrinsic subtype**  Basal  Luminal A  Luminal B  HER2^+^  **Lymph node status**  Negative  Positive  **TP53 status**  Wild type  Mutated  **Pietenpol subtype**  Basal-like 1  Basal-like 2  Immunomodulatory  Mesenchymal  Mesenchymal stem-like  Luminal androgen receptor | 161  387  503  251  548  89  83  130  129  241  611  433  117  313  594  187  111  58  38  100  73  19  83 | 0.54 (0.21-1.35)  0.84 (0.55-1.29)  0.69 (0.50-0.97)  0.74 (0.47-1.18)  0.64 (0.45-0.92)  2.01 (0.75-5.37)  0.42 (0.11-1.70)  1.07 (0.46-2.53)  1.54 (0.75-3.15)  0.68 (0.41-1.12)  0.61 (0.42-0.87)  0.73 (0.50-1.06)  0.48 (0.25-0.93)  0.66 (0.45-0.95)  0.65 (0.44-0.96)  0.59 (0.31-1.15)  0.58 (0.27-1.27)  0.25 (0.07-0.92)  0.83 (0.24-2.86)  1.22 (0.48-3.10)  1.41 (0.64-3.12)  1.35 (0.61-2.97)  0.60 (0.30-1.19) | 0.180  0.440  **0.030**  0.200  **0.014**  0.150  0.210  0.870  0.240  0.130  **0.006**  0.099  **0.027**  **0.026**  **0.031**  0.120  0.170  **0.024**  0.760  0.670  0.390  0.460  0.140 | 345  901  903  801  2061  549  589  800  252  618  1933  1149  251  2020  1133  273  188  171  76  203  177  63  203 | 0.67 (0.39-1.13)  0.80 (0.63-1.01)  0.74 (0.60-0.92)  0.86 (0.69-1.08)  0.70 (0.60-0.83)  0.78 (0.58-1.04)  0.69 (0.49-0.98)  0.66 (0.50-0.86)  0.92 (0.60-1.42)  0.73 (0.57-0.94)  0.72 (0.61-0.85)  0.80 (0.66-0.97)  0.64 (0.43-0.94)  0.72 (0.61-0.85)  0.65 (0.53-0.79)  0.82 (0.54-1.25)  0.77 (0.48-1.24)  0.60 (0.37-0.98)  1.15 (0.57-2.32)  0.92 (0.51-1.66)  0.79 (0.52-1.21)  1.35 (0.61-2.97)  0.84 (0.56-1.26) | 0.130  0.061  **0.008**  0.190  **2.6e-05**  0.089  **0.038**  **0.002**  0.710  **0.014**  **0.000**  **0.024**  **0.023**  **0.000**  **1.8e-05**  0.360  0.280  **0.039**  0.700  0.790  0.280  0.460  0.390 |

## Supplementary Table 5. Correlation of *TXNIP* mRNA expression and clinicopathological factors in breast cancer by Kaplan-Meier plotter database

Abbreviation: HER2, human epidermalgrowth factor receptor-2.

| **Cancer type** | **Dataset** | **Endpoint** | **Number** | **Probe ID** | **Hazard ratio (95% CI)** | **Cox *P*-value** |
| --- | --- | --- | --- | --- | --- | --- |
| **Bladder cancer**  **Brain cancer**  **Breast cancer**  **Colorectal cancer**  **Eye cancer**  Uveal melanoma  **Lung cancer**  Adenocarcinoma  **Skin cancer**  **Soft tissue cancer** | GSE13507  GSE13507  GSE4271-GPL96  MGH-glioma  GSE4412-GPL96  GSE6532-GPL570  GSE6532-GPL570  GSE12093  GSE11121  GSE9893  GSE2034  GSE1456-GPL96  GSE1456-GPL96  GSE1456-GPL96  GSE3494-GPL96  GSE4922-GPL96  GSE17536  GSE14333  GSE22138  jacob-00182-CANDF  GSE19234  GSE30929 | OS  DSS  OS  OS  OS  RFS  DMFS  DMFS  DMFS  OS  DMFS  OS  RFS  DSS  DSS  DFS  DFS  DFS  DMFS  OS  OS  DRFS | 165  165  77  50  74  87  87  136  200  155  286  159  159  159  236  249  145  226  63  82  38  140 | \| ILMN_1697448 \| \| \| --- \| --- \| \| ILMN_1697448 \| \| \| 201010_s_at \| \| \| 31508_at \|  \| \| 201008_s_at \| \| \| 201010_s_at \| \| \| 201010_s_at \| \| \| 201010_s_at \| \| \| 201008_s_at \| \| \| 10372 \|  \| \| 201009_s_at \| \| \| 201009_s_at \| \| \| 201009_s_at \| \| \| 201009_s_at \| \| \| 201010_s_at \| \| \| 201008_s_at \| \| \| 201010_s_at \| \| \| 201010_s_at \| \| \| 201010_s_at \| \| \| 201008_s_at \| \| \| 201009_s_at \| \| \| 201010_s_at \| \| | 0.67 (0.51 - 0.87)  0.58 (0.39 - 0.85)  0.60 (0.36 - 0.99)  0.34 (0.18 - 0.64)  0.53 (0.36 - 0.78)  0.49 (0.30 - 0.81)  0.49 (0.30 - 0.81)  0.42 (0.21 - 0.87)  0.62 (0.45 - 0.86)  1.20 (1.07 - 1.35)  0.54 (0.39 - 0.74)  0.47 (0.30 - 0.74)  0.40 (0.26 - 0.61)  0.38 (0.23 - 0.63)  0.40 (0.23 - 0.71)  0.66 (0.47 - 0.93)  2.57 (1.23 - 5.37)  2.11 (1.21 - 3.69)  0.65 (0.48 - 0.89)  0.50 (0.33 - 0.77)  0.60 (0.40 - 0.90)  0.49 (0.32 - 0.74) | 0.003  0.005  0.046  0.001  0.001  0.005  0.005  0.019  0.004  0.002  0.000  0.001  0.000  0.000  0.002  0.017  0.012  0.009  0.007  0.001  0.013  0.001 |

## Supplementary Table 6. Positive results associated with *TXNIP* expression in different cancers from Prognoscan database

Abbreviation: CI, confidence interval; OS, overall survival; DSS, disease specific survival; RFS, relapse free survival; DMFS, distant metastasis free survival; DFS, disease free survival; DRFS, distant recurrence free survival.

## Supplementary Table 7. Correlation analysis between *TXNIP* and relate genes and markers of immune cells in TIMER2

| **Description** | **Gene markers** | **BRCA (n=1100)** | | | | **LUAD (n=515)** | | | | **KIRC (n=533)** | | | |
| --- | --- | --- | --- | --- | --- | --- | --- | --- | --- | --- | --- | --- | --- |
|  |  | **None** | | **Purity** | | **None** | | **Purity** | | **None** | | **Purity** | |
|  |  | **Cor** | ***P*** | **Cor** | ***P*** | **Cor** | ***P*** | **Cor** | ***P*** | **Cor** | ***P*** | **Cor** | ***P*** |
| CD8+ T cell  T cell (general)  B cell  Monocyte  TAM  M1 Macrophage  M2 Macrophage  Neutrophils  NK cell  Dendritic cell  Th1  Th2  Tfh  Th17  Treg  T cell exhaustion | CD8A  CD8B  CD3D  CD3E  CD2  CD19  CD79A  MS4A1  SDC1  CD14  CD86  FCGR3A  CSF1R  CCL2  CCL5  CD68  INOS (NOS2)  IRF5  CD163  ARG1  MRC1  CEACAM8  ITGAM  CCR7  KIR2DL1  KIR2DL3  KIR2DL4  KIR2DS4  KIR3DL1  KIR3DL2  KIR3DL3  HLA-DPB1  HLA-DQB1  HLA-DRA  HLA-DPA1  CD1C  NRP1  CD11c (ITGAX)   1. bet (TBX21)   STAT1  STAT4  IFN-γ (IFNG)  TNF-α (TNF)  GATA3  STAT6  STAT5A  IL13  BCL6  IL21  CXCL13  STAT3  IL17A  FOXP3  CCR8  STAT5B  TGFβ(TGFB1)  IL2RA  GZMB  HAVCR2  CTLA4  LAG3  PD-1 (PDCD1) | 0.297  0.249  0.259  0.300  0.255  0.217  0.239  0.306  -0.115  0.090  0.128  0.022  0.286  0.098  0.196  0.100  0.105  0.081  0.136  0.132  0.311  -0.001  0.230  0.303  0.032  0.031  -0.007  0.047  0.078  0.106  -0.008  0.316  0.176  0.263  0.314  0.430  0.357  0.174  0.257  0.046  0.333  0.052  0.035  0.162  0.384  0.352  0.014  0.213  0.106  0.075  0.204  -0.011  0.091  0.088  0.397  0.299  0.072  0.053  0.137  0.090  -0.051  0.178 | *******  *******  *******  *******  *******  *******  *******  *******  ******  *****  *******  0.474  *******  *****  *******  ******  ******  *****  *******  *******  *******  0.982  *******  *******  0.293  0.312  0.826  0.117  *****  ******  0.801  *******  *******  *******  *******  *******  *******  *******  *******  0.125  *******  0.085  0.250  *******  *******  *******  0.652  *******  ******  0.013  *******  0.711  *****  *****  *******  *******  0.017  0.079  *******  *****  0.094  ******* | 0.166  0.120  0.107  0.156  0.106  0.079  0.091  0.179  -0.170  -0.024  0.002  -0.067  0.168  -0.025  0.039  -0.023  0.091  0.006  0.032  0.106  0.196  0.006  0.136  0.170  -0.058  -0.041  -0.102  -0.037  0.000  -0.003  -0.064  0.182  0.054  0.131  0.193  0.333  0.279  0.044  0.114  -0.021  0.206  -0.081  -0.027  0.281  0.375  0.281  -0.040  0.205  0.025  -0.037  0.192  -0.087  -0.035  -0.004  0.391  0.202  -0.066  -0.101  0.017  -0.059  -0.159  0.032 | *******  ******  ******  *******  ******  0.012  *****  *******  *******  0.451  0.940  0.035  *******  0.439  0.220  0.461  *****  0.850  0.309  ******  *******  0.856  *******  *******  0.067  0.194  *****  0.239  0.997  0.925  0.045  *******  0.091  *******  *******  *******  *******  0.164  ******  0.515  *******  0.011  0.391  *******  *******  *******  0.203  *******  0.434  0.247  *******  *****  0.265  0.903  *******  *******  0.037  *****  0.589  *****  *******  0.313 | 0.153  0.083  0.241  0.330  0.325  0.169  0.169  0.277  0.266  0.175  0.329  0.240  0.418  0.090  0.175  0.304  0.076  0.343  0.275  0.123  0.495  0.377  0.462  0.415  -0.031  -0.044  -0.144  0.029  -0.025  0.008  -0.062  0.527  0.376  0.497  0.540  0.526  0.294  0.313  0.280  0.104  0.297  -0.023  0.226  0.324  0.306  0.436  0.015  0.234  0.025  0.132  0.161  -0.018  0.275  0.309  0.330  0.419  0.184  -0.096  0.331  0.248  0.033  0.178 | ******  0.059  *******  *******  *******  ******  ******  *******  *******  *******  *******  *******  *******  0.042  *******  *******  0.085  *******  *******  *****  *******  *******  *******  *******  0.485  0.315  *****  0.514  0.575  0.864  0.160  *******  *******  *******  *******  *******  *******  *******  *******  0.018  *******  0.605  *******  *******  *******  *******  0.726  *******  0.569  *****  ******  0.689  *******  *******  *******  *******  *******  0.030  *******  *******  0.452  ******* | 0.094  0.036  0.187  0.292  0.284  0.108  0.112  0.227  0.272  0.122  0.288  0.203  0.382  0.028  0.115  0.272  0.048  0.323  0.240  0.121  0.474  0.369  0.434  0.387  -0.052  -0.091  -0.189  -0.010  -0.060  -0.036  -0.080  0.509  0.339  0.476  0.521  0.499  0.277  0.278  0.241  0.057  0.247  -0.085  0.172  0.277  0.328  0.409  -0.023  0.230  -0.015  0.060  0.158  -0.059  0.231  0.265  0.319  0.391  0.135  -0.175  0.294  0.195  -0.029  0.123 | 0.037  0.423  *******  *******  *******  0.016  0.013  *******  *******  *****  *******  *******  *******  0.534  0.010  *******  0.290  *******  *******  *****  *******  *******  *******  *******  0.249  0.043  *******  0.821  0.185  0.422  0.075  *******  *******  *******  *******  *******  *******  *******  *******  0.203  *******  0.060  ******  *******  *******  *******  0.615  *******  0.741  0.183  ******  0.194  *******  *******  *******  *******  *****  *******  *******  *******  0.521  ***** | 0.088  0.071  0.027  0.073  0.078  -0.055  -0.060  0.119  -0.032  0.010  0.186  0.216  0.230  0.032  -0.031  0.107  0.421  0.081  0.302  0.147  0.410  0.116  0.217  0.074  0.180  0.150  0.016  0.125  0.193  0.158  -0.028  0.240  0.180  0.281  0.281  0.263  0.460  0.090  0.243  0.305  0.187  0.029  -0.016  0.036  0.398  0.208  -0.043  0.346  0.018  -0.094  0.487  -0.039  -0.093  0.086  0.549  0.119  0.178  0.085  0.228  0.085  -0.054  0.002 | 0.042  0.102  0.529  0.092  0.071  0.202  0.165  *****  0.466  0.826  *******  *******  *******  0.458  0.472  0.014  *******  0.062  *******  ******  *******  *****  *******  0.087  *******  ******  0.719  *****  *******  ******  0.522  *******  *******  *******  *******  *******  *******  0.038  *******  *******  *******  0.505  0.712  0.407  *******  *******  0.324  *******  0.678  0.031  *******  0.363  0.031  0.046  *******  *****  *******  0.050  *******  0.049  0.216  0.958 | 0.100  0.082  0.038  0.088  0.093  -0.041  -0.055  0.151  -0.044  0.026  0.210  0.237  0.244  0.039  -0.016  0.098  0.408  0.060  0.293  0.124  0.398  0.102  0.218  0.063  0.163  0.162  0.021  0.129  0.179  0.151  0.004  0.259  0.186  0.297  0.305  0.261  0.450  0.099  0.250  0.305  0.202  0.024  -0.023  0.084  0.372  0.219  -0.007  0.335  0.013  -0.086  0.484  -0.009  -0.084  0.093  0.525  0.127  0.171  0.096  0.213  0.105  -0.037  0.013 | 0.031  0.079  0.421  0.058  0.047  0.376  0.241  *****  0.344  0.579  *******  *******  *******  0.406  0.734  0.035  *******  0.195  *******  *****  *******  0.029  *******  0.176  ******  ******  0.651  *****  ******  *****  0.935  *******  *******  *******  *******  *******  *******  0.034  *******  *******  *******  0.610  0.617  0.071  *******  *******  0.885  *******  0.778  0.065  *******  0.846  0.070  0.047  *******  *****  ******  0.040  *******  0.024  0.433  0.773 |

Abbreviation: BRCA, breast invasive carcinoma; LUAD, lung adenocarcinoma; KIRC, kidney renal clear cell carcinoma; TAM, tumor-associated macrophage; NK cell, natural killer cell; Th, T helper cell; Tfh, Follicular helper T cell; Treg, regulatory T cell. Cor, R value of Spearman’s correlation. None, correlation without adjustment. Purity, correlation adjusted by purity. **P* < 0.01; ***P* < 0.001; ****P* < 0.0001.

# Reference

1. Thul PJ, Lindskog C. The human protein atlas: A spatial map of the human proteome. Protein Sci. 2018;27(1):233-244.
2. Mizuno H, Kitada K, Nakai K, Sarai A. PrognoScan: a new database for meta-analysis of the prognostic value of genes. BMC Med Genomics. 2009;2:18. Published 2009 Apr 24.
